# Metabarcoding reveals high diversity of benthic foraminifera linked to water masses circulation at coastal Svalbard

**DOI:** 10.1111/gbi.12530

**Published:** 2022-10-19

**Authors:** Ngoc‐Loi Nguyen, Joanna Pawłowska, Inès Barrenechea Angeles, Marek Zajaczkowski, Jan Pawłowski

**Affiliations:** ^1^ Institute of Oceanology Polish Academy of Sciences Sopot Poland; ^2^ Department of Earth Sciences University of Geneva Geneva Switzerland; ^3^ Department of Genetics and Evolution University of Geneva Geneva Switzerland

**Keywords:** Atlantic water, benthic foraminifera, metabarcoding, sedimentary DNA, Svalbard

## Abstract

Arctic marine biodiversity is undergoing rapid changes due to global warming and modifications of oceanic water masses circulation. These changes have been demonstrated in the case of mega‐ and macrofauna, but much less is known about their impact on the biodiversity of smaller size organisms, such as foraminifera that represent a main component of meiofauna in the Arctic. Several studies analyzed the distribution and diversity of Arctic foraminifera. However, all these studies are based exclusively on the morphological identification of specimens sorted from sediment samples. Here, we present the first assessment of Arctic foraminifera diversity based on metabarcoding of sediment DNA samples collected in fjords and open sea areas in the Svalbard Archipelago. We obtained a total of 5,968,786 reads that represented 1384 amplicon sequence variants (ASVs). More than half of the ASVs (51.7%) could not be assigned to any group in the reference database suggesting a high genetic novelty of Svalbard foraminifera. The sieved and unsieved samples resolved comparable communities, sharing 1023 ASVs, comprising over 97% of reads. Our analyses show that the foraminiferal assemblage differs between the localities, with communities distinctly separated between fjord and open sea stations. Each locality was characterized by a specific assemblage, with only a small overlap in the case of open sea areas. Our study demonstrates a clear pattern of the influence of water masses on the structure of foraminiferal communities. The stations situated on the western coast of Svalbard that are strongly influenced by warm and salty Atlantic water (AW) are characterized by much higher diversity than stations in the northern and eastern part, where the impact of AW is less pronounced. This high diversity and specificity of Svalbard foraminifera associated with water mass distribution indicate that the foraminiferal metabarcoding data can be very useful for inferring present and past environmental conditions in the Arctic.

## 1 INTRODUCTION

**FIGURE 1:**
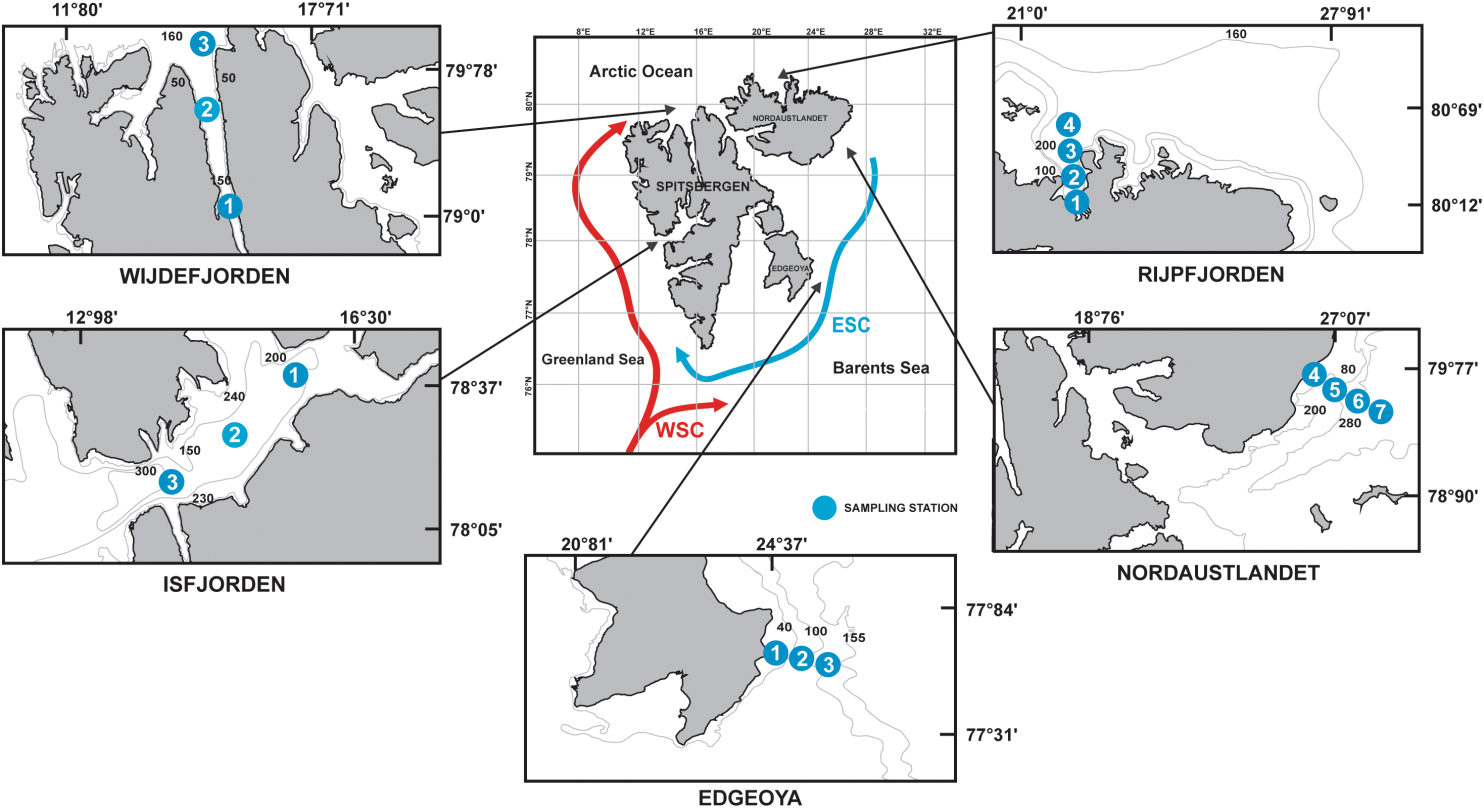
Map showing the location of sampling stations in the fjords of Svalbard Archipelago. These islands include Spitsbergen, Nordaustlandet, and Edgeøya. ESC, East Spitsbergen Current; WSC, West Spitsbergen Current.

The Arctic Ocean is strongly impacted by the increased influence of warm and saline Atlantic water (AW), so‐called “atlantification,” which causes sea ice retreat and sea surface temperature increases (Beszczynska‐Möller et al., 2012; Onarheim et al., 2014; Polyakov et al., 2017), higher input of turbid melt water in summer, restricting the light availability and enhancing flocculation (Nilsen et al., 2008; Zajączkowski et al., 2010) and directly affecting the entire ecosystem of the Arctic (Csapó et al., 2021). The changing environmental conditions in this region introduces a significant impact on shaping biodiversity and the biogeography of many taxonomic groups, such as birds and mammals (Descamps et al., 2017; Vihtakari et al., 2018), fish (Fossheim et al., 2015; Frainer et al., 2017), zooplankton (Grabowski et al., 2019; Hop et al., 2019; Weydmann‐Zwolicka et al., 2021), phytoplankton (Barton et al., 2016; Neukermans et al., 2018), and planktonic foraminifera (Meilland et al., 2020; Ofstad et al., 2021). Such changes in physical drivers lead to a shift in Atlantic species ranges toward the Arctic (Berge et al., 2005), an increase in productivity (Slagstad et al., 2011), and changes in the timing of spring phytoplankton bloom (Zajączkowski et al., 2010). In a marine setting, biotic interactions and physical influences (temperature and salinity) may create shifts in food webs, affecting not only planktonic but also controlling benthic community structure by vertical fluxes of mineral and organic particles or phytoplankton cells to the bottom (Kortsch et al., 2015; Zajączkowski et al., 2010). Particularly, Svalbard ecosystems are currently affected by increased heat transport from the West Spitsbergen Current (WSC) (Dai et al., 2019; Nilsen et al., 2008; Onarheim et al., 2014; Serreze & Barry, 2011), which flows northwards along western Svalbard (Figure 1).

Foraminifera is a group of protists characterized by granuloreticulopodia and belonging to the supergroup of Rhizaria (Burki et al., 2010). Benthic foraminifera are highly abundant and diverse in marine environments from coastal to deep‐sea zones (Gooday & Jorissen, 2012; Murray, 2014; Schoenle et al., 2021), although freshwater forms are also known to exist (Holzmann et al., 2021). Foraminifera typically possesses an organic, agglutinated, or calcareous shell (called test), which readily enter the fossil record, where they are used as index fossils and paleoenvironmental indicators (Murray, 2006, 2014). In the modern, foraminifera are also recognized as important ecological indicators of environmental stress because they are particularly sensitive to abrupt climate change (Kawahata et al., 2019; Prazeres et al., 2017; Wittmann & Pörtner, 2013). It has been demonstrated that the abundance and diversity of benthic foraminifera were extremely variable in the eastern and western Arctic during the last interglacial and glacial climate regimes (Polyak et al., 2013; Wollenburg et al., 2007) and were directly related to changes in sea ice cover, surface productivity, sedimentation, and post‐depositional processes in the Arctic (Backman et al., 2009; Hald & Korsun, 1997; Polyak et al., 2013; Sabbatini et al., 2007).

The traditional approach to analyses foraminiferal diversity consists in sorting and morphological identification of hard‐shelled species belonging to either the class Tubothalamea or Globothalamea, which are commonly larger than 100 μm in size (John W Murray, 2014). However, morphological identification is time‐consuming and taxonomic expertise‐demanding, making it costly and unpractical, particularly for large‐scale surveys. Recently, the metabarcoding of environmental DNA (*e*DNA) samples has provided new insights into the biodiversity and ecological distribution of numerous taxonomic groups and offers an alternative to the traditional morphology‐based approach (Bohmann et al., 2014; Holman et al., 2021; Pawlowski et al., 2021). Metabarcoding consists in high‐throughput sequencing of short DNA barcodes that include enough information for species identification to get a comprehensive inventory of all organisms present in a given sample. For instance, short sequences derived from the 37f hypervariable region of the 18S small subunit (SSU) rRNA gene are widely used in foraminiferal metabarcoding studies (Lejzerowicz et al., 2014; Pawlowski & Lecroq, 2010). To better understand large‐scale patterns of biodiversity and distribution in various groups, this method is increasingly being employed, particularly in marine environments (Armbrecht et al., 2019; Holman et al., 2021; Schoenle et al., 2021; Vargas et al., 2015). Numerous foraminiferal metabarcoding studies were conducted in various coastal areas, e.g. northern Adriatic Sea (Frontalini et al., 2020), Norwegian Sea (Pawlowski et al., 2016), west coast of Scotland (Pawlowski et al., 2014), Dutch Wadden Sea (Chronopoulou et al., 2019), and the deep sea (Cordier et al., 2019; Lecroq et al., 2011; Lejzerowicz et al., 2021; Schoenle et al., 2021). However, the application of *e*DNA metabarcoding to monitor foraminiferal diversity in the Arctic was limited to a few paleogenetic studies using foraminifera as proxies in palaeoceanographic reconstructions and investigating changes in ocean circulation patterns by targeting ancient DNA of non‐fossilized foraminifera from Svalbard (Pawlowska et al., 2014, 2020).

In conventional morphology‐based foraminiferal studies, the sediment samples are sieved before the specimens are sorted (Schönfeld et al., 2012). In all published foraminiferal metabarcoding studies, the DNA was extracted from unsieved sediment samples. This has some benefits, including providing a holistic view of foraminiferal diversity including small‐size species and those lacking the hard shell, potentially reducing sample heterogeneity, detecting mainly small and low abundant taxa, achieving a higher number of reads, or decreasing primer bias due to the reduction in the amount of DNA template produced by the large specimens (Elbrecht et al., 2017; Leray & Knowlton, 2015). Some DNA metabarcoding studies have shown that the preprocessing of samples does not significantly alter metazoan diversity patterns (Brandt et al., 2021; Sinniger et al., 2016). However, the effectiveness of sieving versus non‐sieving in the case of foraminiferal metabarcoding has not been examined yet.

The two main goals of this study are to investigate whether metabarcoding of sieved sediment is effective for the assessment of foraminiferal biodiversity and how the foraminiferal communities respond to rapid environmental shifts in Arctic marine ecosystems. Taxonomic composition, diversity, and distribution of benthic foraminifera were analyzed in fjords and open water areas in Svalbard in order to (1) compare species composition and diversity patterns inferred from sieved and unsieved sediment samples, (2) describe the spatial diversity of Svalbard foraminiferal communities, and (3) identify new potential bioindicators of water mass characteristics.

## 2 STUDY AREA

The Svalbard archipelago is located north of the shallow and productive Barents Sea. The largest island is Spitsbergen, followed by Nordaustlandet and Edgeøya. Approximately 60% of the archipelago is covered by glaciers. The coastline featured numerous fjords, islets, and skerries.

The oceanography of Svalbard region is shaped mainly by the interplay between warm and saline AW and cold Arctic water (ArW), as well as locally formed water masses (Cottier et al., 2005; Hop et al., 2019). AW is transported northward along the Spitsbergen shelf edge as the WSC (Figure 1) (Blindheim & Østerhus, 2005; Loeng, 1991). WSC is one of the major heat contributors to the Arctic Ocean (Spielhagen et al., 2011), transporting heat from low latitudes into the Arctic and transferring it to the atmosphere and adjacent water masses (Saloranta & Haugan, 2004). Between 78 and 80°N, the WSC bifurcates into an eastern (Svalbard) branch and a western (Yermak) branch (Aagaard et al., 1987). The Svalbard Branch flows northeasterly, staying close to the continental margin of Svalbard (Aagaard et al., 1987). The Yermak Branch streams northwards and further recirculates southward as the Return Atlantic Current (Bourke et al., 1988). The Svalbard area is also under the influence of cold ArW that is transported from the north‐eastern Barents Sea by the East Spitsbergen Current (ESC, Figure 1), also called Sørkapp Current or the Coastal Current (Sternal et al., 2014). Mixing of ArW and AW results in the formation of transformed Atlantic water (TAW) which expanded across the shelf and penetrated the fjords (Cottier et al., 2005; Nilsen et al., 2016).

Isfjorden (IS) and Wijdefjorden (WIJ) are located on the west coast of Spitsbergen, along the main pathway of AW inflow (Figure 1). Both fjords are linked directly to shelf and slope areas (Kowalewski et al., 1990; Nilsen et al., 2008) and therefore, their oceanographic conditions are shaped mainly by the inflow of AW and TAW. Isfjorden is considered to be the most AW‐impacted fjord of Spitsbergen (Nilsen et al., 2016). Rijpfjorden (RIJ) is a north‐facing fjord, located on the northern coast of Nordaustlandet. The oceanography of Rijpfjorden is dominated by cold ArW, with a less pronounced impact of AW. However, episodic inflows of AW may occur in ice‐free periods. As such, it is considered to be a typical Arctic fjord. Most of the year, Rijpfjorden is covered by sea ice and/or drifting ice packs (Ambrose Jr. et al., 2006).

The southeastern Nordaustlandet (NAL) and the eastern Edgeøya (EDG) are strongly impacted by the presence of large ice caps, making them one of the largest glacierized areas of Svalbard (Dowdeswell et al., 1986). The tidewater cliffs supply the surrounding areas with large amounts of turbid meltwater (Julian A. Dowdeswell & Bamber, 1995). Water masses around Nordaustlandet and Edgeøya are dominated by ArW, carried by the ESC. However, in periods of strong WSC activity, the presence of AW is also pronounced (Knies et al., 2007).

## 3 MATERIAL AND METHODS

### 3.1 Sampling

The samples were collected at 15 sampling stations from five localities on Western, Northern, and Eastern sides of the Svalbard Archipelago (Figure 1), including three fjord sites (Isfjorden, Wijdefjorden, Rijpfjorden) and two open marine areas in front of tidewater glaciers (Edgeøya, Nordaustlandet). Sampling station coordinates and sampling depths can be found in Table S1. Surface sediment samples were collected with the use of a box corer during the cruise of R/V *Oceania* in August 2016. The upper 2 cm of sediment has been sampled from the surface of approximately 50 cm^2^. Samples for sedimentary *e*DNA analysis were split into two: one half remained unsieved and the other half has been wet sieved on 500, 100, and 63 μm sieves. A fraction smaller than 63 μm was retained. Samples were transferred to sterile containers and frozen at −20°C. In each sampling station, physical properties of the water column from a vertical conductivity‐temperature‐depth (CTD) profiler were obtained using a Mini CTD Sensordata SD202 at intervals of 1 s. Water temperature was reported in degrees celsius (°C), and turbidity was presented in Formazine Turbidity Units (FTU). Water masses were classified according to Cottier et al. (2005). Table S2 contains detailed information.

### 3.2 Metabarcoding analyses

The genomic DNA from size fractions >500, 500–100, and 100–63 μm was extracted from 0.25 g of sediment sample with DNeasy PowerSoil Kit (Qiagen, Hilden, Germany). Approximately 10 g of unsieved part and the remained sediment fraction <63 μm were extracted using DNeasy PowerMax Soil Kit (Qiagen, Hilden, Germany). In total, five amplicon libraries per station were prepared, corresponding to the fractions >500, 500–100, 100–63, and < 63 μm, as well as unsieved samples.

The foraminifera‐specific 37f hypervariable region of 18S rRNA gene was PCR amplified with the primers s14F1/s15 (Barrenechea Angeles et al., 2020; Lejzerowicz et al., 2014), tagged with unique sequences of 8 nucleotides appended at 5′ ends (Esling et al., 2015). The lengths of amplified products are approximately 180 base pairs on average including the specific primers and the tags. Primer sequences and PCR conditions are detailed in Table S3. For each sample, 3 PCR replicates were obtained. PCR products were visualized by 1.5% agarose gel electrophoresis and quantified with Qubit 3.0 fluorometer (Thermo‐Fisher Scientific Inc., Waltham, MA, USA). The PCR products were pooled in an equimolar mix with each duplicate located in a different pool to reach a total quantity of 100 ng of DNA. The pool was purified with High Pure PCR Cleanup Micro Kit (Roche Diagnostics GmbH, Mannheim, Germany). Library preparation was performed with TruSeq® DNA PCR‐Free LT Library Prep Kit (Illumina Inc., San Diego, CA, USA) and was loaded onto a MiSeq instrument for a paired‐end HTS run of 2 × 150 cycles using a v2 kit.

### 3.3 Data quality control and processing

Bioinformatics analyses were performed using the web application SLIM (https://trtcrd.github.io/SLIM) (Dufresne et al., 2019). The reads were first demultiplexed using the double tag demultiplexing algorithm based on their unique barcode sequences. The software package DADA2 (Callahan et al., 2016) was used for quality trimming and filtering sequences, de‐replicating sequences, inferring amplicon sequence variants (ASVs), merging of forward and reverse sequences, and detection and removal of chimeras. Subsequently, all the resulting ASVs tables were curated with the LULU algorithm (Froslev et al., 2017) to remove erroneous ASVs following the online tutorial (https://github.com/tobiasgf/lulu) with default parameters. Final quality filtering of ASVs involved the removal of unique (occurring in only one sample) and rare ASVs (having <10 reads).

The remaining ASVs were compared to the curated database of foraminiferal 18S rDNA sequences (Holzmann & Pawlowski, 2017; Pawlowski et al., 2013) and the PR2 database v4.11.1 (Guillou et al., 2013) using VSEARCH, implemented in SLIM, and BLASTN (Altschul et al., 1990) based on minimum similarity (−perc_identity 80%) and minimum coverage (−qcov_hsp 80%) for the taxonomic assignment to six taxonomic levels (phylum; class; order; family; genus; species). The representative sequences of ASVs that remained unclassified with the foraminiferal database were aligned in a stand‐alone BLAST using BLAST (v2.7.1) search against the NCBI's non‐redundant nucleotide database. The sequences diverging by less than 1% were considered as belonging to the same species/genus. ASVs below 99% identity were classified at the family, order, or class or as unassigned foraminifera. Finally, taxonomic compositions in terms of cluster abundance were compared among processing methods only using clusters reliably assigned at the species/genus level.

### 3.4 Statistical analysis

Before statistical analyses, the ASV table was filtered to remove ASVs that were classified as planktic or non‐foraminifera. For each sample, datasets of four size fractions were combined as a sieved dataset and compared to an unsieved dataset in further analysis. All statistical analyses were performed in R, version 4.1.0 (R Core Team, 2018). All formal hypothesis tests were conducted on the 5% significance level (α = .05).

To compare the community composition among methods and size fractions, Venn diagrams were constructed using the *venn* package (Dusa, 2018). The ASVs rarefaction curves were calculated to visualize whether or when a plateau was reached based on the number of eventually retained ASVs and reads using the iNEXT package (Hsieh et al., 2016). The species accumulation curve was also created using the function *specaccum* in the *vegan* package (Oksanen, 2007). The data of each sample were normalized using the cumulative sum scaling method available on the metagenome‐Seq Bioconductor package (Paulson et al., 2013). Based on the normalized data, four alpha diversity indexes and non‐metric multidimensional scaling (nMDS) on the Bray–Curtis similarity coefficient to analyze differences in the beta diversity of the community composition were calculated with the *vegan* package (Oksanen, 2007). We used the *pheatmap* package (Kolde, 2019) to create a heatmap based on Spearman's correlation. The influences of environmental factors were calculated with the *envfit* function. A global one‐way analysis of similarities (ANOSIM), permutational multivariate analysis of variance (PERMANOVA), and method to fit environmental vectors onto ordination were computed using the function *anosim*, *adonis*, and *envfit* with 999 permutations and the Bray–Curtis distance matrix to test whether there were significant differences in community composition among methods and locations of sampling units.

Finally, sparse partial least squares (sPLS) regression, available in the *mixOmics* package (Le Cao et al., 2008; Rohart et al., 2017), was used for the multivariate analysis of the combined foraminiferal datasets at ASVs level to identify ASVs that were more predictive of the observed environmental response. Pairwise similarity matrices of an sPLS model with 2 components were computed and displayed by the function *cim*. This approach enabled us to identify high correlations between certain ASVs and environmental parameters.

## 4 RESULTS

### 4.1 CTD data

**FIGURE 2:**
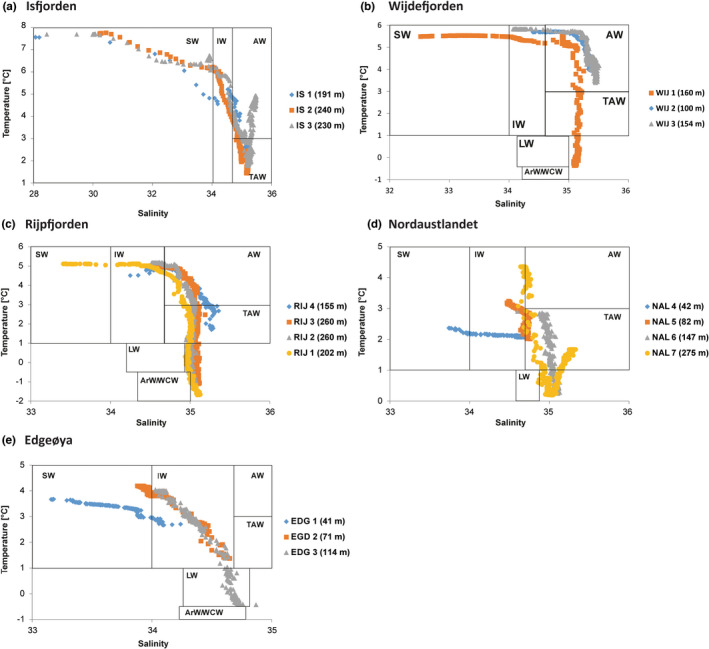
Temperature (°C) and salinity in Svalbard stations: (a) Isfjorden—IS, (b) Wijdefjorden—WIJ (c) Rijpfjorden—RIJ, (d) Nordaustlandet—NAL, and (e) Edgeøya—EDG. Values in brackets are water depth at the time of sampling. ArW, Arctic water; AW, Atlantic water; IW, intermediate water; LW, local water; SW, surface water; TAW, transformed Atlantic water; WCW, winter cooled water. Water masses are classified after Cottier et al. (2005).

Temperature, salinity, and turbidity for all sampling stations are presented in Figures 2 and S1, respectively. AW and TAW dominated the water masses in Isfjorden (Figure 2a) and Wijdefjorden (Figure 2b), which have the highest temperatures and salinities of the investigated stations. Additionally, surface water (SW) and intermediate water (IW) were recorded at all stations in Isfjorden and station WIJ1. In Isfjorden, the highest temperature of 7.7°C was observed at the surface and progressively decreased toward the bottom to 1.4°C. Similarly, the temperature fluctuated from 5.8°C at the surface to −0.4°C near the bottom of Wijdefjorden. Water temperatures above 0°C were noted up to 111 m in depth at the station WIJ1 and in the whole water column at other stations. Salinity was the lowest at the surface, reaching 28.1 in Isfjorden and 32.5 in Wijdefjorden, respectively. The lowest salinity was recorded at inner Isfjorden and Wijdefjorden (IS1 and WIJ1) and the highest values near the mouth of these fjords (IS3 and WIJ3). The turbidity increased from the inner fjord (0.1 FTU) toward the fjord's mouth (particularly up to 12.5 FTU) in Isfjorden, reaching its maximum in the SW at the station IS3. In contrast, turbidity was the highest in the surface layer and decreased from the inner fjord toward the fjord mouth, ranging from 5.2 to 0.1 FTU.

Rijpfjorden was characterized by the lowest near‐bottom temperatures (as low as −1.7°C) among the studied fjords (Figures 2c and S1). Three sites (RIJ1, RIJ2, and RIJ3) reported cold and saline winter cooled water (WCW) in addition to other water masses (AW, TAW, SW, and IW). The temperature ranged from 5.2°C at the top to −1.7°C near the bottom, with the salinity varying from 33.4 to 35.4 throughout the water mass. The lowest near‐bottom temperature was noted at the station RIJ1. The water temperature of the whole water column was observed to be above 0°C at station RIJ4. Turbidity reached over 12.5 FTU at the station RIJ 3 in the near‐bottom water layer and decreased to 0.1 FTU toward the mouth of the fjord.

In the region of eastern Svalbard, the Nordaustlandet stations were generally under influence of TAW, whereas AW was noted only at the glacier‐distant station NAL7 (Figure 2d). NAL4 was the sole station where neither TAW nor AW was detected. SW and IW were other water masses recorded near the Nordaustlandet. Water temperature oscillated between 4.4°C at the surface to 0.2°C near the bottom. Salinity at the surface ranged from 33.6 to 35.3 and increased toward the glacier‐distant stations. The water column had relatively low turbidity (<1 FTU). The only exception was glacier‐proximal station NAL4, where turbidity reached 54.8 FTU, which was the highest value of all studied sites.

Edgeøya stations were the most distinct locations, with the absence of Atlantic‐origin waters (Figure 2e). The IW was detected at all stations, while local water (LW) occurred only at station EGD3. Toward the bottom of the stations, the temperature in the water column varied between 4.2 and − 0.5 and salinity ranged from 33.2 to 34.9. The highest turbidity was recorded at EGD1; it increased with depth to reach 48.8 FTU near the bottom. At the other stations, turbidity values oscillated from 0.3 to 4.3 FTU.

### 4.2 Metabarcoding data

We obtained a total of 5,968,786 raw paired‐end reads. After bioinformatic processing, the numbers of the raw reads were reduced to 5,579,202 with 4,836,419 in a sieved dataset, and 742,783 in an unsieved dataset. The number of reads per sample is indicated in Table S4. One sample, unsieved IS2, produced a low number of reads and was not included in the analysis of foraminiferal diversity. After LULU curation step and strict filtering of ASVs, 1384 ASVs (1354 ASVs of sieved and 1053 ASVs of unsieved samples) representing 5,483,500 reads (98.28% of the total reads count) were retained for downstream analysis (Table S5). The average numbers of sequences per station were 317,306 for the sieved and 51,976 for the unsieved datasets.

**FIGURE 3:**
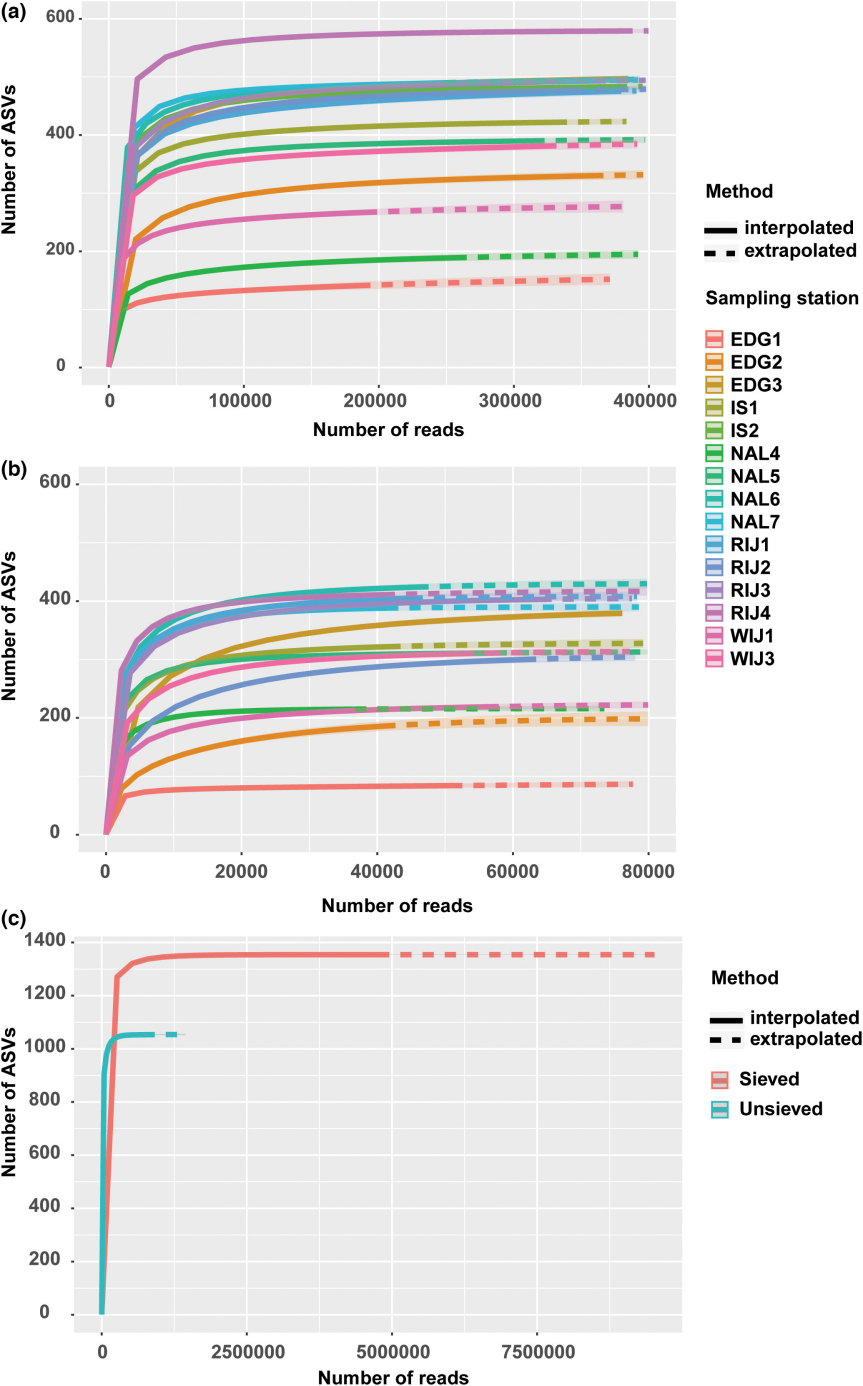
Rarefaction curves showing the relationship between sequencing depth and species richness in amplicon sequence variant (ASVs) of the 37f libraries from 15 stations of individual sieved samples (a), unsieved samples (b) and combining of sieved and unsieved samples (c). The solid line is the rarefaction curve based on the abundance of observed reads, and the dotted line is the extrapolation curve based on the abundance of extrapolated reads.

The rarefaction curves were plotted at the sample level based on the number of retained ASVs and reads (Figure 3). The rarefaction curves showed that the filtered ASV datasets reach saturation levels, indicating that most of the diversity had been captured and allowing for richness comparison among samples for all individual stations of each location (Figure 3a,b) and both methods (Figure 3c). The species accumulation curves of the samples for each location increase with the number of samples, indicating that the existing sample size could meet the needs of this study (Figure S2). Considering the accumulation of ASVs richness across two datasets, sieved datasets exhibited a higher saturation degree, respectively, and the species richness of WIJ, IS, and RIJ stations is higher than the individual station of EDG and NAL.

### 4.3 Taxonomic composition of foraminiferal metabarcodes

Overall, the retained sequences were assigned to 1384 foraminiferal ASVs. Among them, 758 ASVs were assigned to the class Monothalamea, 252 ASVs were assigned to the class Globothalamea, and only 14 ASVs were assigned to the class Tubothalamea (Table S5). The 360 ASVs, classified as Foraminifera_X, had low similarity levels and could not be assigned to any existing clades. More than half of the ASVs (51.73%) were assigned with low similarity (<0.9).

**FIGURE 4:**
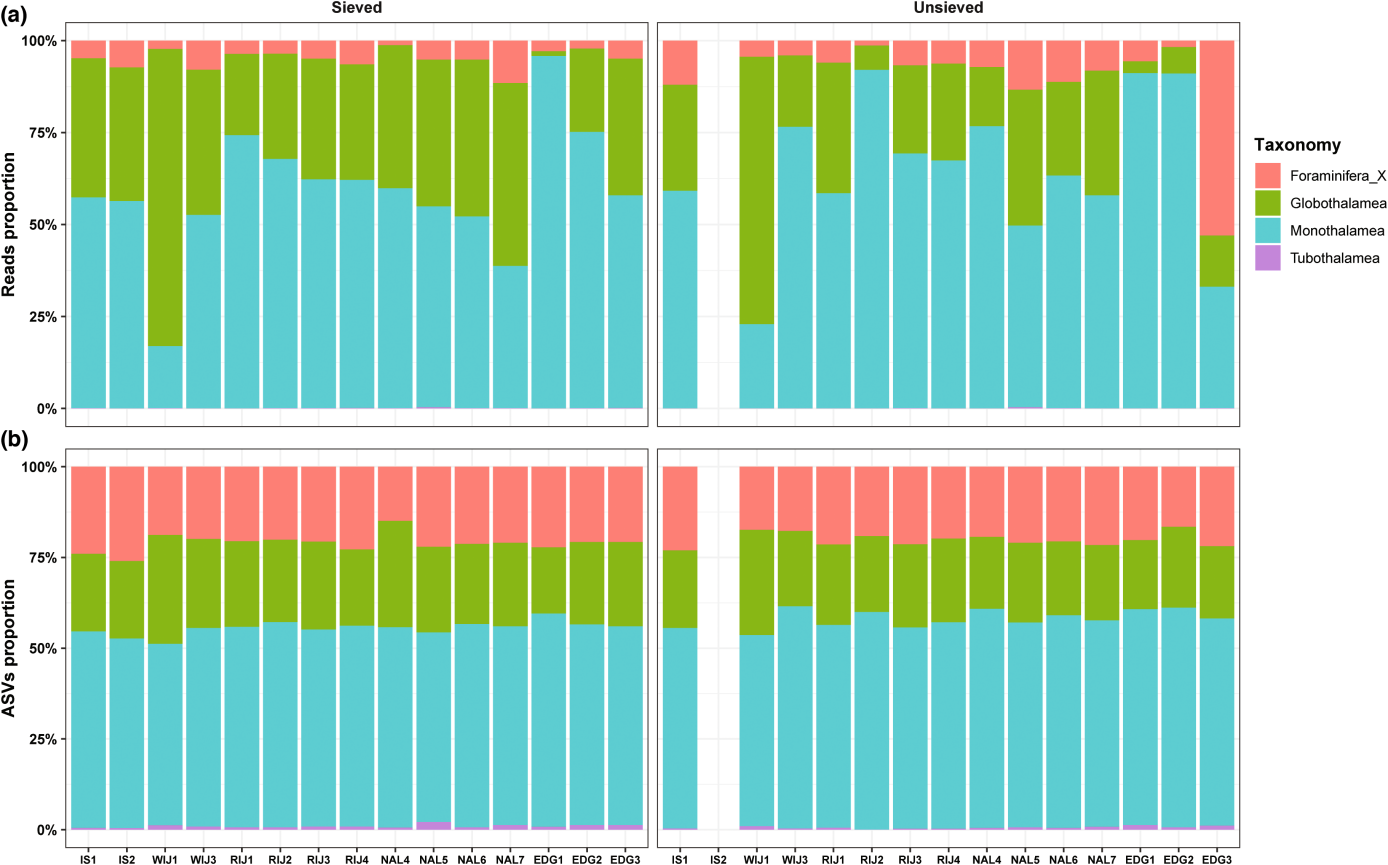
Proportions of reads (a) and ASVs (b) assigned to different foraminiferal classes detected in sieved and unsieved samples at different sites.

**FIGURE 5:**
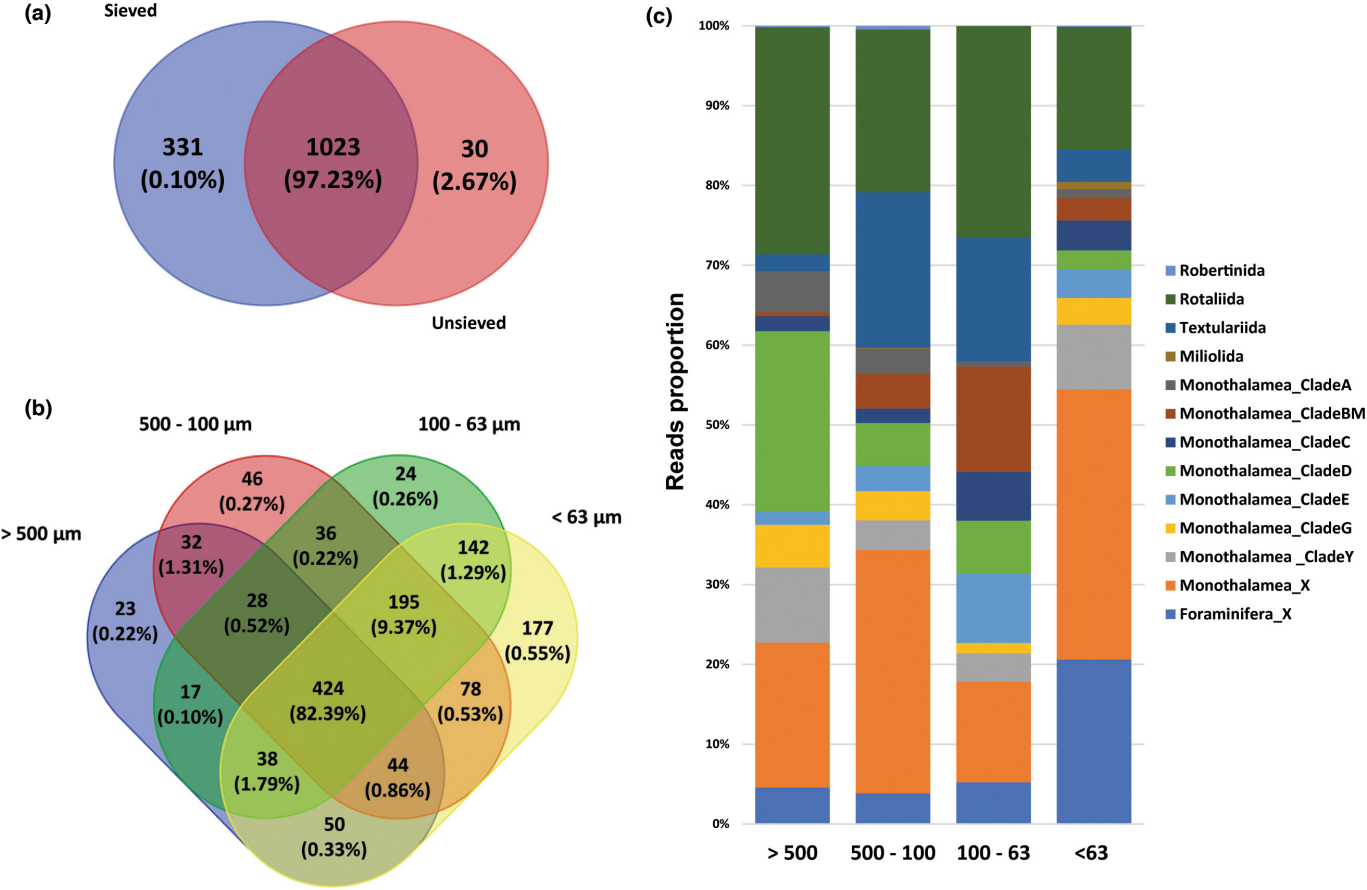
Venn diagrams showing the shared and unique numbers ASVs and proportion of reads for the sieved (combined fractions) and unsieved samples (a), and between different size fractions (b). Bar plots represent the proportion of reads assigned to the taxonomic composition of each fraction by order/clade (c).

The sieved and unsieved sediment DNA samples resolved comparable communities at the class level (Figure 4). Pairwise comparisons indicated no overall significant differences in community composition between sieved and unsieved datasets (ANOSIM statistic R < 0, *p* > .05 and PERMANOVA, Table S6). In Figure 5a, the Venn diagram showed that 1023 ASVs (corresponding to 97.23% of the reads) were shared among sieved and unsieved samples. The sieved dataset had 331 unique ASVs, while unsieved dataset comprised only 30 unique ASVs (corresponding to 0.1% and 2.67% of the reads, respectively).

Taxonomic composition in sieved samples noticeably changed between size fractions (Figures 5b,c). The <63 μm fraction comprises 1151 ASVs, corresponding to 83% of ASVs (Figure 5b). It also recovered more unique ASVs (177, corresponding to 13.07% of ASVs) than any other fractions. Shared foraminiferal ASVs among fractions including 424 ASVs (corresponding to 82.39% of the reads), mostly belonged to Monothalamea (59.72%), and Globothalamea (Rotaliida 25.17%, Textulariida 10.60%) as shown in Figure S3. In all fractions (Figure 5c), the monothalamous taxa made up from 58% to 80% of reads. Non‐described monothalamiids dominated in the 500–100 μm and < 63 μm fractions (30.48% and 33.85% of reads, respectively). For multichambered globothalamids, order Textulariida accounted for 15%–20% of the reads in 500–63 μm fractions, while Rotaliida represented more than 20% of reads in >63 μm fractions. Interestingly, most reads of unique ASVs were assigned to specific foraminiferal groups in each fraction, e.g., Foraminifera_XX (70.69%) in >500 μm fraction, Rotaliida (66.21%) in 500–100 μm fraction, Textulariida (39.28%) in <63 um fraction, and Clade Y of Monothalamea (70.24%) in 100–63 μm fraction, see Figure S3.The taxonomic composition of benthic foraminifera also changed between the locations. At the class level (Figure 4), the monothalamous taxa were the dominant group which accounted for an average of 56.06% and 61.77% of total ASVs and reads in both datasets, respectively. The highest proportion of monothalamiids (95.80%) was observed at the station EDG1 in the sieved dataset. The contribution of monothalamiids decreased in the deeper EDG stations in favor of the class Globothalamea. Comparatively, the average proportions of ASVs and reads assigned to Globothalamea were 22.60% of the ASVs and 30.75% of reads, respectively. The highest relative abundance of Globothalamea (80.80%) occurred in the WIJ3_Sieved sample. The Tubothalamea represented only a minor part of the total community (0.06% of ASVs and 7.42% of reads on average).

**FIGURE 6:**
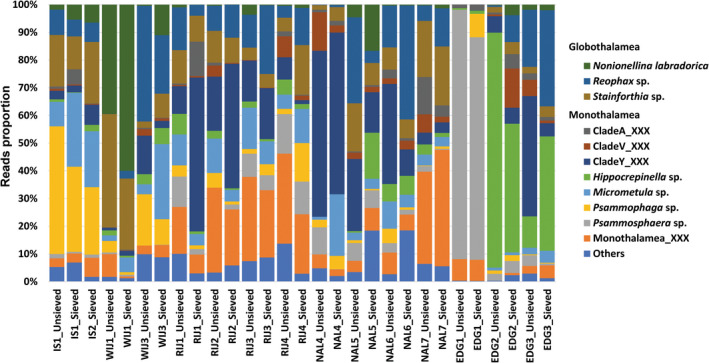
Patterns of relative abundance of dominant genera and species in the sieved (combined fractions) and unsieved sediment samples for each location.

The variations of foraminiferal assemblages between different sampling localities were also reflected in the taxonomic composition of foraminiferal assemblages at the lower taxonomic level. To compare species composition in each station, all ASVs which had an identity percentage with the reference database of more than 99% and no <10 reads were picked and those attributed to the same taxa were merged (Figure 6).

In the stations located at the western coast of Spitsbergen (IS, WIJ), foraminiferal communities were dominated by genera *Psammophaga* and *Micrometula*, which together made up to 57.35% of reads. The WIJ1 was the only station where the majority of sequences belonged to a textularid *Reophax* sp. and a rotalid *Stainforthia* sp. Another rotalid species *Nonionellina labradorica* was present at all IS and WIJ stations, mainly distributed in WIJ1 (sieved: 59.99%, unsieved: 39.46%), WIJ3_Sieved (11%), and IS2_Sieved (6.5%). The northern stations (RIJ) were dominated by monothalamiids assigned to Clade Y and Monothalamea_XXX. At the outermost station RIJ4, also higher percentages of *Psammophaga* sp. and *Psammosphaera* sp. sequences occurred. At the stations located at eastern Svalbard (NAL), the number of Clade Y sequences decreased toward the glacier‐distant stations, while the percentage of Monothalamea_XXX increased. Also, glacier‐distal stations were characterized by a higher proportion of Globothalamea, mainly *Reophax* sp. and *Stainforthia* sp. The foraminiferal community in EDG1 was dominated by a monothalamid *Psammosphaera* sp. (up to 90%), while *Hippocrepinella* sp. dominated in EDG2 and EDG3 (from 11% to 84%). Also, the percentage of *Reophax* sp. sequences increased toward the glacier‐distal stations, from <1% to reaching up to 34.68% at the station EDG3_Sieved.

### 4.4 Alpha and beta diversity patterns

#### 4.4.1 Alpha diversity

**FIGURE 7:**
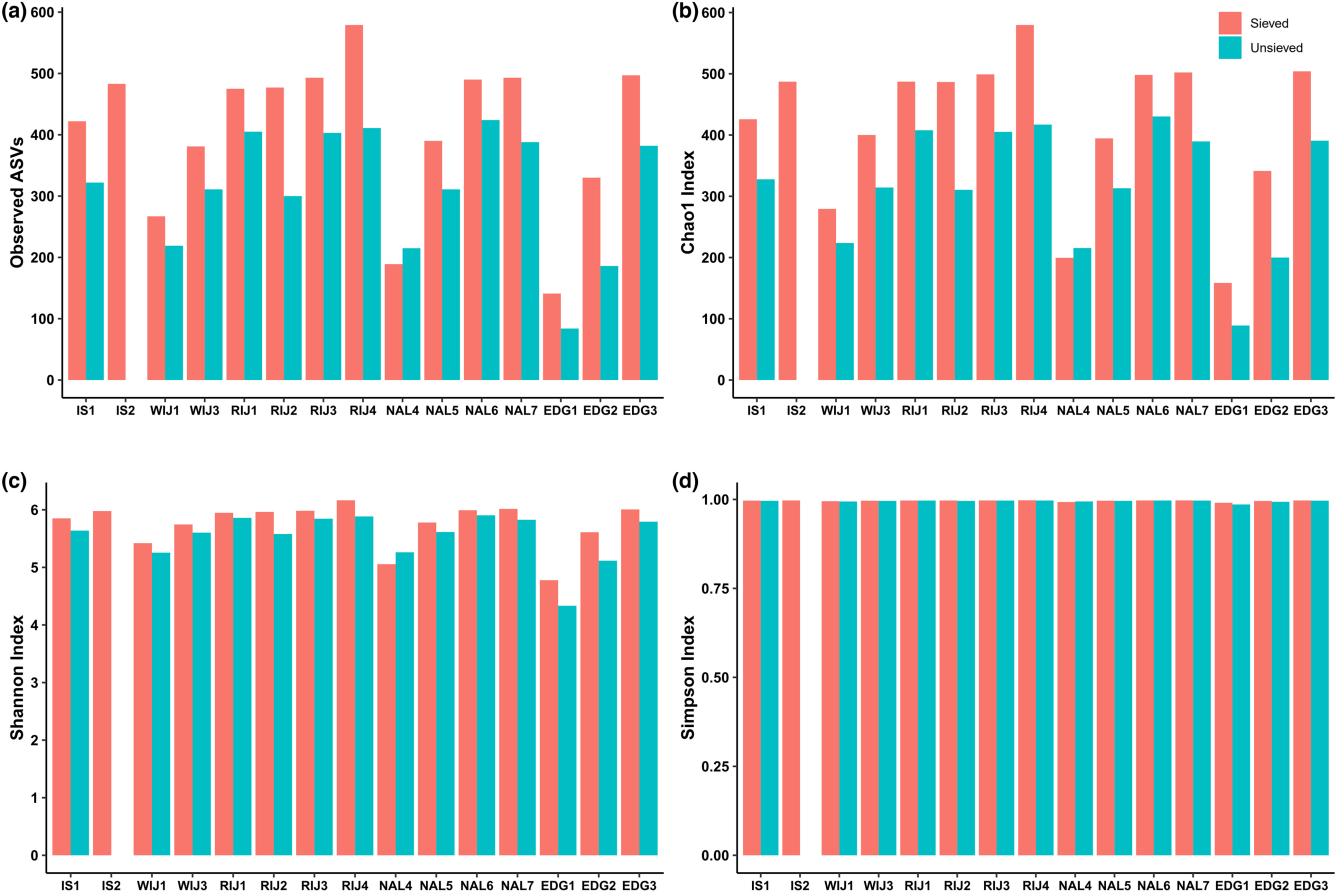
Bar plots of alpha diversity indices, including community richness (a: Observed ASVs, b: Chao1) and diversity (c: Shannon, d: Simpson) for the 15 sieved samples and 14 unsieved samples using retained ASV read abundances.

The four alpha diversity indices (Observed ASVs, Chao1, Simpson and Shannon) were measured separately for sieved and unsieved datasets (Figure 7) and showed clear variation between different locations. On the one hand, the number of ASVs varied substantially depending on sample treatments and locations. In terms of sample treatment, sieved samples recovered higher Observed ASVs and Chao1 indices (Figure 7a,b), but not Simpson or Shannon (Figure 7c,d). On the other hand, the measured alpha diversity indices tended to increase with increasing distance from the glacier. In general, the alpha diversity indices of the fjords (IS, WIJ, RIJ) were higher than those of open sea areas (NAL, EDG), indicating higher foraminiferal diversity in fjords.

#### 4.4.2 Beta diversity

**FIGURE 8:**
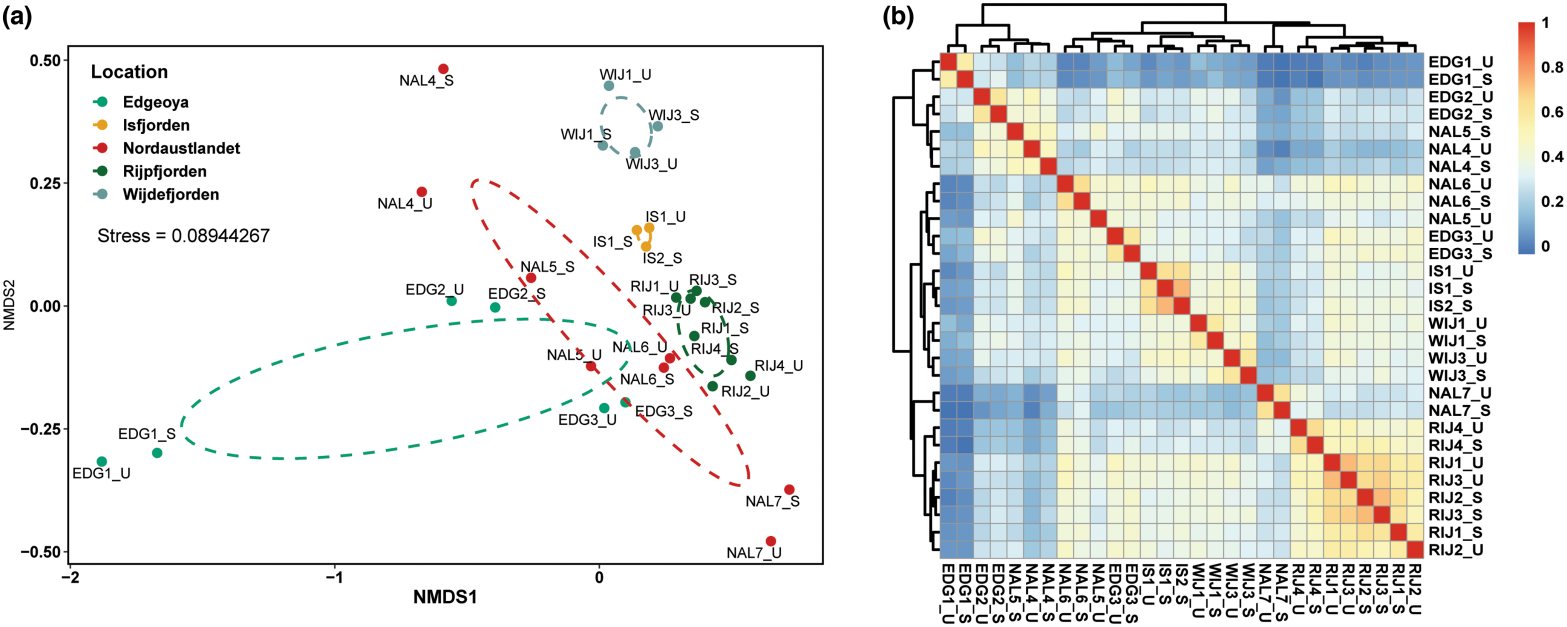
Community structuring of benthic foraminifera using nonlinear multidimensional scaling based on Bray–Curtis distance similarity coefficient (a) and heatmap based on Spearman's correlation coefficient for fractions and unsieved samples (b). Stress value is displayed on the plot.

The nMDS and heatmap of all stations (Figure 8) supported the findings of the ordinances, showing that foraminiferal communities detected in the sieved samples differed from those detected in the unsieved samples, but not significantly (Table S7). The nMDS and heatmap patterns also revealed the spatial distribution of the foraminiferal communities among the different localities. In Figure 8a, the nMDS analyses produced a similar pattern with sieved and unsieved datasets, although community segregation was observed in ordinations of EDG and NAL sites. The communities were distinctly separated between fjord stations and opened sea stations, as shown by low‐stress values. Although the fjord samples formed tight clusters, the samples from each fjord were not overlapping with the samples from other fjord locations. On the contrary, the communities obtained from EDG and NAL sites formed clusters with much larger internal compositional differences and have an overlap between the two sites. Heatmap further clarified the community structuration with the stations and datasets (Figure 8b), which were not visible on the nMDS (except for NAL5). The sampling sites were grouped in two main clusters: cluster 1 aggregating 3 stations (EDG1, EDG2, NAL4) and cluster 2 comprising the 12 remaining stations that were grouped into three subclusters. The stations of two fjords (IS and WIJ) had homogeneous communities and formed one separate subcluster. Two other subclusters are formed by (i) NAL6, NAL5, EDG3, and (ii) all RIJ stations and NAL7.

### 4.5 sPLS prediction analysis

**FIGURE 9:**
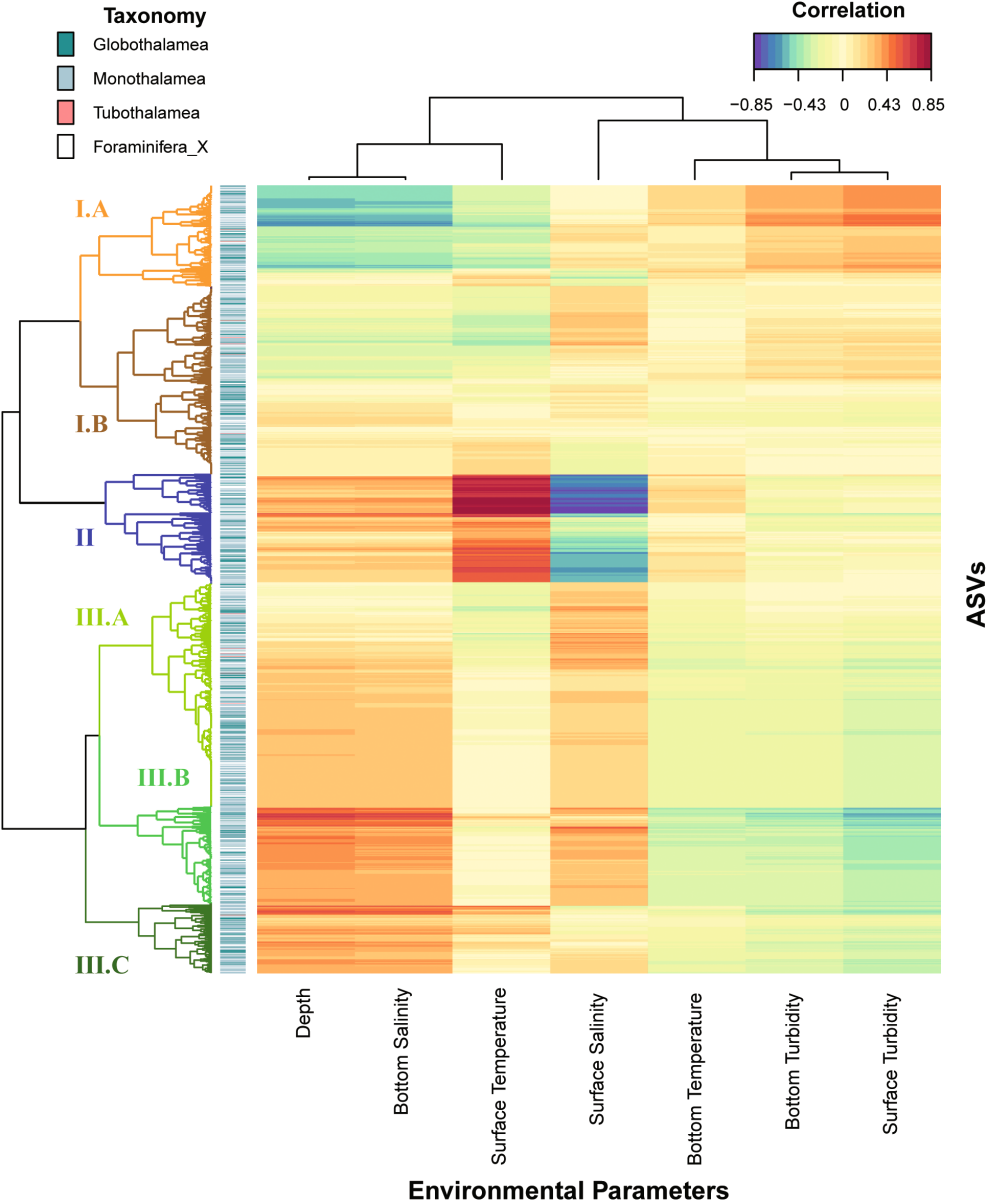
Clustered image map (CIM) of the first two sPLS dimensions, displaying pairwise correlations between foraminiferal ASVs of combined unsieved and sieved samples associated with environmental parameters. Correlations between ASVs and environmental parameters are depicted as a clustered heat map (detailed results in Table S8). Red and blue indicate positive and negative correlations, respectively.

The results of the sPLS regression allowed the detection of several foraminiferal ASVs lineages for which relative sequence abundance was correlated with environmental parameters (Figure 9 and Table S8). The sPLS regression and subsequent hierarchical clustering suggested that the data were separated into three clusters (Figure 9). These include lineages identified as potential indicators of water mass characteristics.

In cluster I.A, the ASVs exhibited a positive correlation with turbidity and negative correlation with factors such as depth, the salinity of bottom water, and temperature of the SW, with the ASVs predominantly affiliated as members of monothalamiids: *Hippocrepinella* sp. (ASV3, ASV10, ASV21), *Psammosphaera* sp. (ASV6), *Saccamminidae* sp. (ASV12), CladeY_spallogJAP (ASV22), STICKY_ICE (ASV39), *Pelosinella fusiformis* (ASV82), and globothalamids: *Buliminella* sp. (ASV14), *Nonionellina labradorica* (ASV20), *Cibicides* sp. (ASV91). The ASVs within cluster II revealed a strong and positive correlation with temperature as well as a negative correlation with salinity in the SW masses. This cluster included globothalamids: *Stainforthia* sp. (ASV1), *Virgulinella fragilis* (ASV79), *Reophax sp*. (ASV92*), Cibicidoides fletcheri* (ASV23), and monothalamiids: *Psammophaga* sp. (ASV25, ASV34, ASV42, ASV53, ASV64), *Micrometula* sp. (ASV4), ENFOR2_EnvHabIC19 (ASV45), CladeA (ASV85, ASV32). Some ASVs belonging to cluster II also had a strong positive correlation with the depth and bottom water salinity (Table S8).

Additionally, we observed positive correlations with depth and salinity in clusters III.A and III.B. Most of ASVs belonging to these clusters were classified as undetermined Monothalamea: Monothalamea_XX (39 ASVs), ENFOR XX (20 ASVs), CladeG (19 ASVs), CladeTIN (16 ASVs). In terms of ASV abundance, the dominant ASVs included environmental monothalamiids (ASV11, ASV44, ASV81, ASV66, ASV93, ASV88, ASV69, ASV78), CladeC_spsaccam (ASV59), *Gloiogullmia* sp. (ASV63), *Cibicides* sp. (ASV38), *Nonionella auris* (ASV31), rotalid (ASV96), *Reophax* sp. (ASV41, ASV13, ASV18), *Stainforthia* sp. (ASV77). Cluster III.C had positive associations with the depth, the bottom water salinity, and the surface temperature. ENFOR2_XXX (ASV408, ASV136, ASV125) exhibited their highest abundances in this cluster.

## 5 DISCUSSION

### 5.1 Is pre‐sieving useful for foraminiferal metabarcoding?

The methodological aim of this study was to compare the results of metabarcoding analyses based on sieved and unsieved sediment samples. Sieving is a common procedure in the conventional microscopic study of foraminiferal assemblage analyzing mainly hard‐shelled, multi‐chambered taxa preserved in fixed and dried sediment samples (Schönfeld et al., 2012). In contrast, metabarcoding studies of unsieved sediment samples usually provide a foraminiferal assemblage dominated by poorly known, soft‐walled, or naked monothalamous taxa (Lecroq et al., 2011; Pawlowski et al., 2014). Because of this, it is difficult to compare the results of traditional morphology‐based studies with those of metabarcoding analysis, which provide very different types of data (Frontalini et al., 2020).

As shown by our study, the taxonomic composition differed between the fractions. For example, the order Rotaliida was the most abundant in 500–100 and 100–63 μm size fractions. Also, another hard‐shelled order Textulariida, which is microscopically studied in the 500–100 μm fraction, in metabarcoding data is present mainly in fractions 500–100 and 100–63 μm (Figure 5c). This is congruent with the rotaliids and textulariids dominating microscopic assemblage found in >63 μm sieved fraction. On the other hand, the smallest fraction (<63 μm) was dominated by monothalamiids and undetermined Foraminifera (Figure 5c), which may suggest the presence of some unknown, tiny monothalamous species. Compared to morphological approaches, DNA‐based metabarcoding provides a more holistic picture of foraminiferal communities, including tiny species present in <63 μm fraction as well as those that are not preserved in dried material used in conventional surveys.

We also observed some differences between sieved and unsieved samples regarding the alpha diversity. The total number of recovered ASVs was clearly higher in sieved than in unsieved samples (approximately 30% ASVs). However, this could be explained by the difference in the number of DNA extraction, PCR amplification, and sequencing depth. In the case of sieved samples, the datasets included four DNA extractions, one for each size fraction, while only one DNA extraction was performed for non‐sieved sediment samples. Further, sieving probably reduces PCR inhibitors as well as non‐targeted taxa in the samples. In total, the number of sequences obtained for sieved fractions was several times higher, allowing for the detection of higher diversity in sieved compared to unsieved samples. However, no significant difference between the sieved/unsieved samples was observed in alpha diversity measures such as Shannon's and Simpson's that take abundance and evenness of the sample into consideration as shown in Figure 7. Although sieving might have been predicted to lead to a reduction in alpha diversity due to the loss of microfauna and extracellular debris, this has not been observed in previous studies (Brannock & Halanych, 2015; He et al., 2020). In addition, nMDS of the beta diversity matrices and correlation test showed sieved and unsieved samples clustered together (Figure 8, Table S7), indicating that there is no significant difference in community composition inferred by the two methods.

To conclude, the decision of whether the sediment samples should be sieved or not shall be based on the type of questions one wants to answer with metabarcoding data as well as the composition and characteristics of initial samples. Sieving of samples destinated for metabarcoding analysis might be useful if particular groups of foraminifera are targeted (e.g., Rotaliida, Textulariida), for example, to compare with microscopic studies or to identify some tiny species present in fine size fractions. In general, the unsieved samples provide a more complete overview of the taxonomic composition of the foraminiferal community. However, as shown by our study, both metabarcoding datasets reveal similar trends in foraminiferal diversity. Size sieving might have some advantages; however, it also has some drawbacks, as (i) it is time‐consuming, (ii) requires higher volume samples, and (iii) there is a possibility of cross‐contamination between samples. Therefore, either extracting DNA directly from sediment or after sieving should be carefully considered when evaluating foraminiferal communities across metabarcoding studies.

### 5.2 Distribution patterns of foraminifera in Svalbard

The most striking result of this study is the variations of foraminiferal assemblage between different sampling localities. The taxonomic composition of foraminiferal communities is generally specific to each location (Figure 7). Each fjord forms a separate cluster (IS, WIJ, and RIJ) and only some stations at open‐water areas overlap with each other (EDG and NAL) as Figures 7 and 8. However, there are some similar trends documented at different locations, such as the high proportion of monothalamiids in near‐glacier settings (Figure 6) or the increase of alpha diversity from glacier proximal/inner to glacier‐distant/outer stations (Figure 7), which are in agreement with the previous morphology‐based studies (Hald & Korsun, 1997; Majewski et al., 2005; Sabbatini et al., 2007).

The high‐Arctic settings are usually considered as a cold system influenced at different levels by ArW during summer to late autumn (Wallace et al., 2010), and covered by sea ice in winter (Ambrose Jr. et al., 2006; Dai et al., 2019; Leu et al., 2011). However, the increased influence of AW and winter sea ice loss is observed in recent years (Dahlke et al., 2020; Nilsen et al., 2008; Pavlova et al., 2019). We speculate that hydrographic conditions would lead to isolating populations from different settings and creating unique structures of the foraminiferal community.

It is well known that the unique habitats of fjords can support a high diversity and distinct biological communities (Gooday et al., 2005; Hald & Korsun, 1997; Majewski et al., 2005; Sabbatini et al., 2007; Walseng et al., 2018; Włodarska‐Kowalczuk et al., 2013). Fjords create a variety of habitats suitable for specific species, where many species can converge and reach high population densities. Western Spitsbergen fjords are among the most AW‐impacted areas. Both Isfjorden and Wijdefjorden are directly connected to the slope and shelf areas, which enables AW penetration into the fjords. Moreover, Isfjorden stations are located in the central basin of the fjord, which resulted in limited glacial influence. This led to the formation of foraminifera communities characterized by a relatively high proportion of globothalamids and the presence of monothalamiids community dominated by the genera *Psammophaga* and *Micrometula* (Figure 6). Similar distribution patterns were previously observed in west Spitsbergen fjords (Majewski et al., 2005; Sabbatini et al., 2007). Only station WIJ1 displayed a unique structure with a clear dominance of Rotaliida (Figures 4 and 6). Station WIJ1 is located in the inner fjord, close to the glacier termini and is influenced by turbid meltwater runoffs. The dominance of Rotaliida contradicts previous studies, indicating that glacier proximal settings are dominated by monothalamous foraminifera (Pawłowska et al., 2016; Sabbatini et al., 2007). However, this distribution pattern could be also explained by the natural patchiness of foraminiferal distribution.

In the northern site (RIJ), the dominant component of foraminiferal assemblages was undetermined monothalamiids (Figures 4 and 6). The dominance of these small, soft‐walled species was previously observed in areas characterized by close to the glacier‐proximal zone and influenced by freshwater inputs (Pawlowska et al., 2014; Sabbatini et al., 2007). Northern Svalbard in general and Rijpfjorden in particular are considered to be a typical Arctic setting, where sea ice forms in autumn and lasts until summer. Also, the drifting ice pack is often transported to the fjord during the summer (Ambrose Jr. et al., 2006), which leads to the formation of cold and saline WCW. This process may create a more ideal environment in inner fjords where monothalamiids thrive (Gooday et al., 2005; Korsun & Hald, 1998; Sabbatini et al., 2007).

Foraminiferal communities of open water areas (eastern Svalbard) have generally lower diversity and form different groups compared to those from western Svalbard fjords. Stations from the regions of Nordaustlandet and Edgeøya are located in front of large tidewater glaciers, releasing large amounts of turbid meltwater (Figure S1). However, only Nordaustlandet was influenced by AW and TAW, while the Edgeøya oceanographic conditions were shaped mainly by LW masses. These led to the creation of distinctly different foraminiferal communities. NAL stations were characterized by a wide range of undetermined monothalamiids, while the EDG stations were dominated by a few monothalamous species representing genera *Hipocrepinella* and *Psammosphaera* (Figure 6). Both genera were previously recorded in shallow‐water assemblages of Spitsbergen fjords (Gooday et al., 2005; Majewski et al., 2005). In particular, station EDG1 exhibited a unique foraminiferal community, composed almost exclusively of *Psammosphaera* sp.

### 5.3 Influence of AW on foraminifera community

The responses of benthic foraminifera to alterations in temperature and salinity in the water column are common and include expansions or retractions of distribution ranges or changes in assemblage compositions (Dong et al., 2019; Langer et al., 2013; Weinmann & Goldstein, 2016). We hypothesize that the composition of foraminiferal communities in our data resulted from water mass conditions. This hypothesis is strengthened by the clear clustering of the community in groups corresponding to different oceanographic regimes, in which stations from regions impacted by AW and/or sea ice clustered separately.

As shown by the nMDS plot and heatmap (Figure 8), the separation between two main clusters has a strong relationship with the characteristics of water masses. Cluster 1 comprises exclusively the stations of the eastern part of the archipelago (EDG1, EGD2, NAL4, NAL5), characterized by colder, and less salty water, associated with turbid glacial meltwater (Meslard et al., 2018). On the contrary, Cluster 2 includes mainly stations from the western and northern part of Svalbard where the impact of warmer and more saline AW was much more pronounced, as confirmed by our CTD profile (Figure 2). Also, subclusters that formed within cluster 2 reflected different impacts of AW. The first subcluster comprises stations (NAL6, EDG3) located in the glacial‐distant regions of Nordaustlandet and Edgeøya, influenced mainly by TAW. The second subcluster (IS1, IS2, WIJ1, WIJ3) includes the most AW‐impacted stations located on the western coast of the archipelago, while the third subcluster (NAL7, RIJ1, RIJ2, RIJ3, RIJ4) is composed of stations located in north‐eastern Svalbard, influenced both by the inflow of AW and WCW.

The increased AW inflow, higher light availability, and the decline of sea ice around Svalbard affect the primary productivity, changing both the timing of phytoplankton bloom and phytoplankton community structure. This may have significant effects on food web dynamics, affecting higher trophic levels, including benthic communities (Csapó et al., 2021). On the other hand, recent model projections indicated low mean habitat loss of benthic macrofauna under recent climate changes, which questions the vulnerability of Arctic benthos to atlantification (Renaud et al., 2019). This stands in clear opposition to the morphological observations that testate foraminifera communities from Svalbard fjords revealed significant changes, both in terms of abundance and species composition, related to the influence of AW (Kujawa et al., 2021). Our study confirms the impact of AW on foraminiferal communities, suggesting that AW is one of the primary factors shaping the benthic foraminifera assemblages and thus foraminifera may be potential indicators of atlantification.

Through sPLS analysis of combining datasets, we identified foraminiferal taxa that could become potential bioindicators of “atlantification.” This group of species includes some monothalamiids belonging to genera *Psammophaga* and *Micrometula* as well as some undetermined monothalamous species belonging to environmental lineage ENFOR2 and Clade A. The genera *Psammophaga* and *Micrometula* are widespread in many coastal areas including polar regions (Altin‐Ballero et al., 2013; Gooday et al., 2011) and are considered as bioindicator candidates in several studies (Pawlowski et al., 2014; Smith & Goldstein, 2019). However, the limited knowledge about the ecology of those taxa, as well as lacking information on their distribution in the Nordic Seas precludes making any general conclusions. Among potential bioindicators, there are also some globothalamids, such as *Stainforthia* sp., *Virgulinella fragilis*, *Reophax* sp., and *Cibicidoides fletcheri*. *Cibicidoides fletcheri* is common in the North Atlantic (Dorst & Schönfeld, 2013), but to the best of our knowledge, it was not recorded in Svalbard before. One of the major signs of increasing inflow of AW (so‐called atlantification) is the northward shift of boreal species, the trend observed in the case of zooplankton, fish, and benthic organisms (Csapó et al., 2021). A recent morphological study of Svalbard foraminifera revealed the presence of boreal species *Melonis affinis* in the northern part of the archipelago (Kujawa et al., 2021). *Reophax* sp. and *Stainforthia* sp. are commonly found in Svalbard (Hald & Korsun, 1997). Also, species belonging to the genus *Reophax* are considered as indicators of AW (Majewski et al., 2009). The agreement of our results with previous morphology‐based studies further proves the potential use of foraminiferal metabarcoding in biomonitoring studies.

Apart from indicators of atlantification, we identified monothalamiids that show a strong correlation with turbidity, but not with depth or salinity. These species included various monothalamids assigned to *Hippocrepinella, Psammosphaera*, *Saccamminidae*, as well as the CladeY_spallogJAP, and several unassigned STICKY_ICE ASVs. This correlation is particularly strong in stations closer to the coast, which is probably caused by enhanced turbidity due to sediment‐laden meltwater plumes. All these monothalamids were previously recorded in Svalbard (Gooday et al., 2005; Majewski et al., 2005; Pawłowska et al., 2016) and were abundantly sequenced in the settings characterized by a high level of environmental disturbance, suggesting that they are highly resistant to environmental disturbance (Pawlowski et al., 2014). Moreover, morphological studies reported *Hippocrepinella* sp., *Psammosphaera* sp., *Saccamminidae* sp., in the shallow‐water parts of the fjords, located close to meltwater outflows (Gooday et al., 2005; Majewski et al., 2005; Sabbatini et al., 2007). These findings underline how important can be to include soft‐walled monothalamous foraminifera in metabarcoding studies to enhance limited knowledge about their ecology for potential use in biomonitoring.

## 6 CONCLUSIONS

This study is the first to use high‐throughput sequencing to comprehensively analyze the foraminiferal communities within marine sediments from Svalbard, which provide better knowledge of foraminiferal diversity and distribution patterns in the Arctic's fjords. The DNA sequencing results from sieved and unsieved sediment revealed a high diversity of the Svalbard foraminifera compared to traditional morphology‐based studies and variation in the taxonomic composition of foraminiferal communities from five sampling areas. Foraminiferal diversity and species richness increased from glacier proximal/inner to glacier‐distant/outer stations and were higher in the fjords than in the open water. Moreover, the structure of foraminiferal community is clearly influenced by different water masses, with a particular impact of AW in the Svalbard region. Numerous potential molecular foraminiferal bioindicators for water mass characteristics were identified. This should be confirmed by analysing more samples from reference areas in North Atlantic. With the increasing numbers of metabarcoding studies, the impact of climate warming and associated oceanographic changes on Arctic benthic communities could be better assessed and expanded to those organisms that are not covered by the conventional morphological approach.

## CONFLICT OF INTEREST

The authors declare no competing interests.

## Supporting information


**FIGURE S1** Water temperature [°C], salinity, and turbidity [FTU] measured at the sampling stations at the time of sampling in August 2016. (A) IS—Isfjorden, (B) WIJ—Wijdefjorden, (C) RIJ—Rijpfjorden, (D) NAL—Nordaustlandet, and (E) EDG—Edgeoya. Water masses are classified after Cottier et al.^1^ (see Table S2).
**FIGURE S2** Species accumulation curve for the 15 samples, obtained with the function *specaccum* of the “*vegan*” package; it shows the relationship between observed ASVs and number of samples. The lines indicate the averaged accumulated increase of detected OTUs vs. number of samples (1000 bootstrap sampling replicates). The shadowed area indicates the standard deviation. The continuous line represents all the samples pooled together.
**FIGURE S3** Taxonomic composition of unique and shared foraminiferal ASVs in different size fractions. Bar plots represent the proportion of reads assigned to the taxonomic composition of each fraction by order/clade.
**TABLE S1** Location of the sampling stations
**TABLE S2** Definition of water masses in Svalbard according to Cottier et al.^1^
**TABLE S3** Primers and PCR thermal cycling profile^2,3^ used in this study
**TABLE S4** Sequencing data statistics and quality control results
**TABLE S5** Total number of ASVs and sequence read abundance identified within the foraminiferal 37f dataset
**TABLE S6** Permutational multivariate analysis of variance (PERMANOVA) results based on Bray–Curtis dissimilarities using *adonis* procedure for showing the effects of method, site, depth, and their interaction on the foraminiferal community structure
**TABLE S7** Correlation of environmental variables with foraminiferal community compositions following *envfit* procedure. Pr (>r) indicates the level of significance based on 999 random permutations. Significance codes: 0 ‘***’, .001 ‘**’, .01 ‘*’
**TABLE S8** Foraminiferal ASVs and their correlations as computed using sparse PLS regression (sPLS). Correlations between foraminiferal ASVs and environmental parameters are revealed using standard methods for regression‐based modelling of high dimensional data. Environmental parameters correspond to depth, bottom salinity, surface temperature, surface salinity, bottom temperature, bottom turbidity, and surface turbidity.

## Data Availability

All raw sequencing reads have been deposited in the NCBI Short Read Archive (SRA) database under Bioproject accession number PRJNA768352.
