# Supplementary material for: Metabarcoding reveals high diversity of benthic foraminifera linked to water masses circulation at coastal Svalbard
Source: Geobiology. 2022 Oct 19;21(1):133–50. doi: 10.1111/gbi.12530 (PMC10092302; doi:10.1111/gbi.12530)
Supplement: Supplementary file 1 — FIGURE S1 Water temperature [°C], salinity, and turbidity [FTU] measured at the sampling stations at the time of sampling in August 2016. (A) IS—Isfjorden, (B) WIJ—Wijdefjorden, (C) RIJ—Rijpfjorden, (D) NAL—Nordaustlandet, and (E) EDG—Edgeoya. Water masses are classified after Cottier et al.1 (see Table S2). FIGURE S2 Species accumulation curve for the 15 samples, obtained with the function specaccum of the “vegan” package; it shows the relationship between observed ASVs and number of samples. The lines indicate the averaged accumulated increase of detected OTUs vs. number of samples (1000 bootstrap sampling replicates). The shadowed area indicates the standard deviation. The continuous line represents all the samples pooled together. FIGURE S3 Taxonomic composition of unique and shared foraminiferal ASVs in different size fractions. Bar plots represent the proportion of reads assigned to the taxonomic composition of each fraction by order/clade. TABLE S1 Location of the sampling stations TABLE S2 Definition of water masses in Svalbard according to Cottier et al.1 TABLE S3 Primers and PCR thermal cycling profile2,3 used in this study TABLE S4 Sequencing data statistics and quality control results TABLE S5 Total number of ASVs and sequence read abundance identified within the foraminiferal 37f dataset TABLE S6 Permutational multivariate analysis of variance (PERMANOVA) results based on Bray–Curtis dissimilarities using adonis procedure for showing the effects of method, site, depth, and their interaction on the foraminiferal community structure TABLE S7 Correlation of environmental variables with foraminiferal community compositions following envfit procedure. Pr (>r) indicates the level of significance based on 999 random permutations. Significance codes: 0 ‘***’, .001 ‘**’, .01 ‘*’ TABLE S8 Foraminiferal ASVs and their correlations as computed using sparse PLS regression (sPLS). Correlations between foraminiferal ASVs and environmental parameters are revealed using [file GBI-21-133-s001.zip › GBI_12530_Nguyen-et-al-supplementary-information (1).docx]

**Supporting Information for**

**Metabarcoding reveals high diversity of benthic foraminifera linked to water masses circulation at coastal Svalbard**

**This file includes:**

Figures S1 to S3

Table S1 to S8

SI References

**Supplementary Figures and Tables**

**Figure S1.** Water temperature [°C], salinity and turbidity [FTU] measured at the sampling stations at the time of sampling in August 2016. (A) IS – Isfjorden, (B) WIJ – Wijdefjorden, (C) RIJ – Rijpfjorden, (D) NAL – Nordaustlandet, and (E) EDG – Edgeoya. Water masses are classified after Cottier, et al. ^1^ (see Table S2).


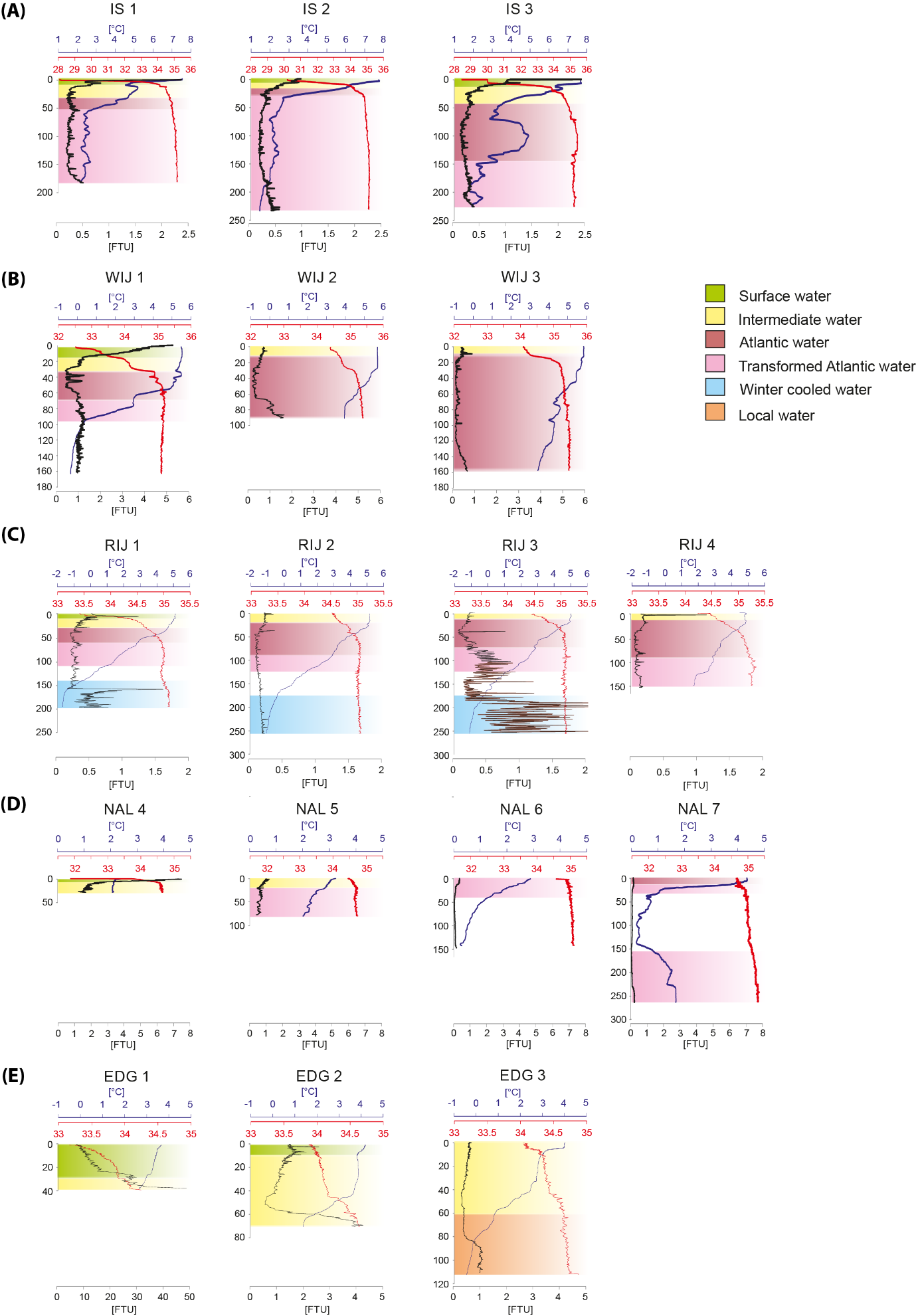


**Figure S2.** Species accumulation curve for the 15 samples, obtained with the function *specaccum* of the “*vegan*” package; it shows the relationship between observed ASVs and number of samples. The lines indicate the averaged accumulated increase of detected OTUs vs. number of samples (1,000 bootstrap sampling replicates). The shadowed area indicates the standard deviation. The continuous line represents all the samples pooled together.


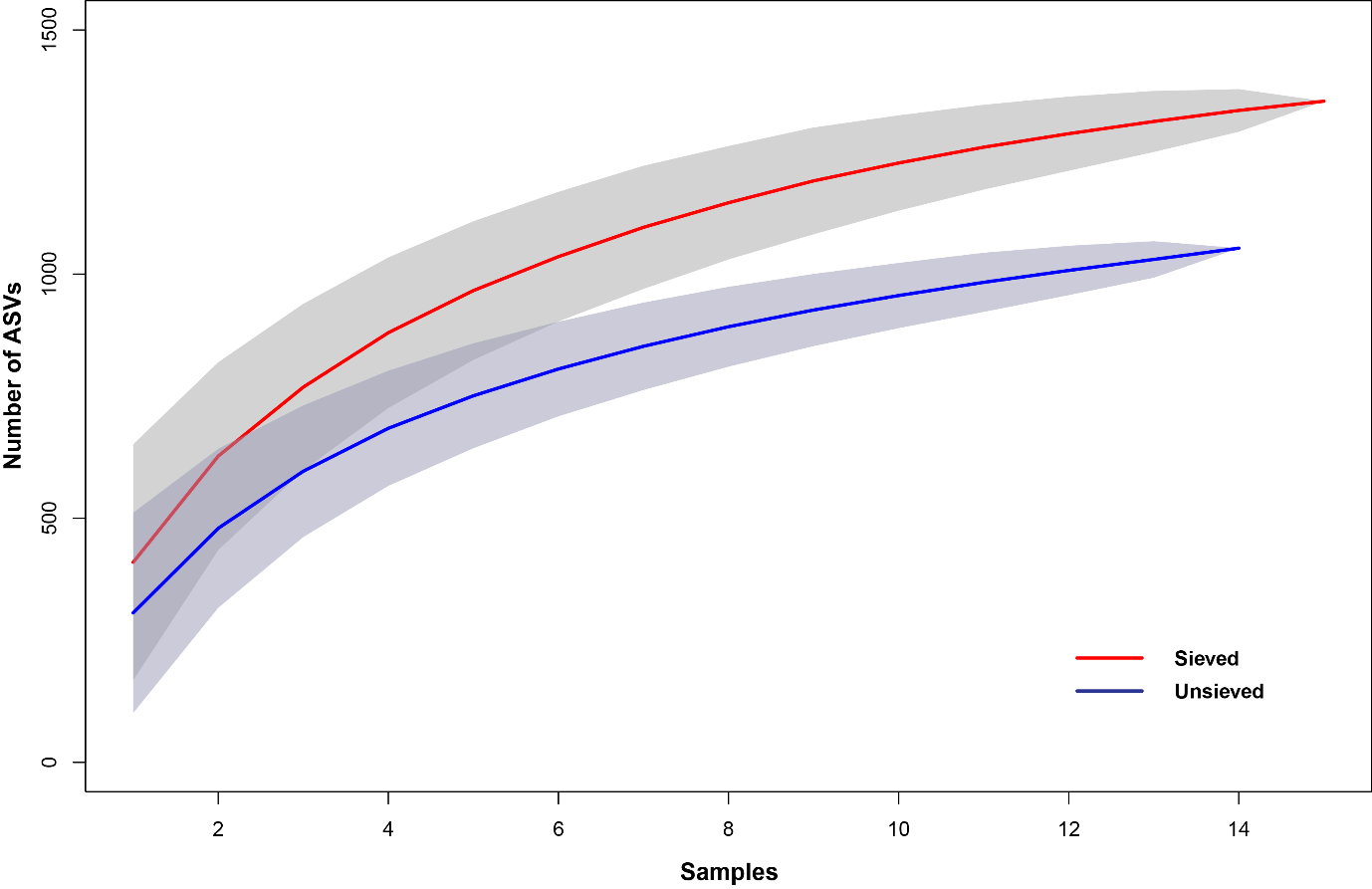


**Figure S3.** Taxonomic composition of unique and shared foraminiferal ASVs in different size fractions. Bar plots represent the proportion of reads assigned to the taxonomic composition of each fraction by order/clade.


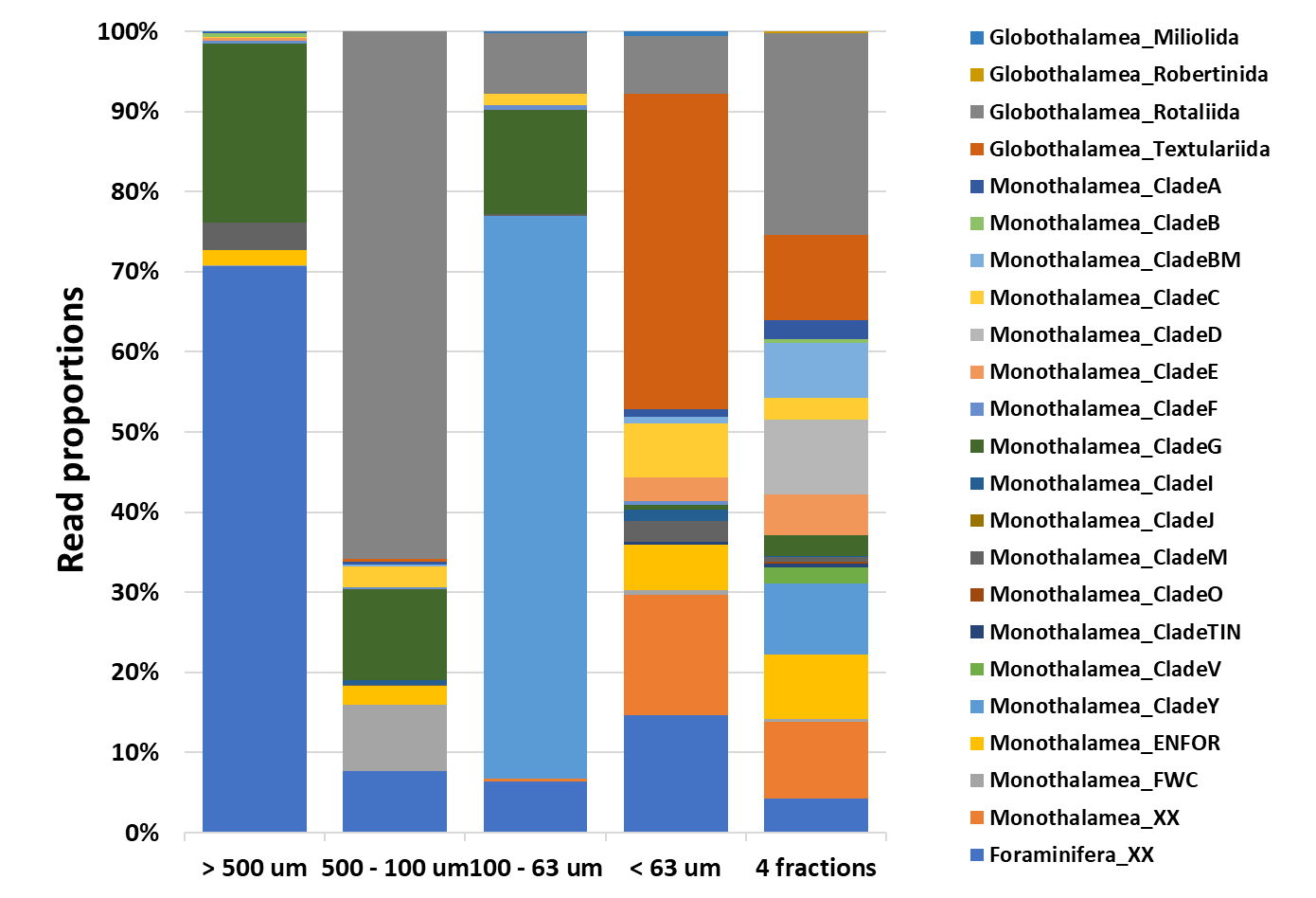


**Table S1.** Location of the sampling stations.

| Station | Depth (m) | Latitude | Longitude |
| --- | --- | --- | --- |
| Edgeoya 1 | 41 | 77°42'55.5"N | 24°12'51.2"E |
| Edgeoya 2 | 71 | 77°41'08.8"N | 24°39'23.0"E |
| Edgeoya 3 | 114 | 77°39'45.1"N | 25°00'09.7"E |
| Isfjorden 1 | 191 | 78°24'00.0"N | 15°35'41.4"E |
| Isfjorden 2 | 240 | 78°15'53.4"N | 14°51'12.0"E |
| Isfjorden 3 | 230 | 78°09'48.0"N | 14°07'06.0"E |
| Nordaustlandet 4 | 42 | 79°42'43.6"N | 26°34'57.3"E |
| Nordaustlandet 5 | 82 | 79°40'30.4"N | 26°48'57.1"E |
| Nordaustlandet 6 | 147 | 79°36'14.5"N | 27°30'11.8"E |
| Nordaustlandet 7 | 275 | 79°33'11.1"N | 28°00'20.8"E |
| Rijpfjorden 1 | 202 | 80°05'22.4"N | 22°12'59.8"E |
| Rijpfjorden 2 | 260 | 80°18'27.8"N | 22°10'57.1"E |
| Rijpfjorden 3 | 260 | 80°22'21.2"N | 22°05'44.2"E |
| Rijpfjorden 4 | 155 | 80°30'17.3"N | 22°02'59.8"E |
| Wijdefjorden 1 | 160 | 79°09'27.8"N | 16°00'05.1"E |
| Wijdefjorden 2 | 100 | 79°29'21.5"N | 15°33'28.5"E |
| Wijdefjorden 3 | 154 | 79°53'48.9"N | 15°19'04.0"E |

**Table S2.** Definition of water masses in Svalbard according to Cottier, et al. ^1^.

| Water masses | | Temperature (°C) | Salinity |
| --- | --- | --- | --- |
| Atlantic water | AW | > 3.0 | > 34.65 |
| Arctic water | ArW | -2.5 | 34.3 - 34.8 |
| Transformed Atlantic water | TAW | 1.0 - 3.0 | > 34.65 |
| Winter Cooled Water | WCW | < -0.5 | 34.4 - 35.0 |
| Surface water | SW | > 1.0 | < 34.0 |
| Local water | LW | -1.5 | 34.3 - 34.85 |
| Intermediate water | IW | > 1.0 | 34.0- 34.65 |

**Table S3:** Primers and PCR thermal cycling profile ^2,3^ used in this study.

| Target gene and amplicon size | 37f SSU rDNA, ca. 180 bp | | |
| --- | --- | --- | --- |
| Primer name and sequence * | s14F1 (5′- XXXXXXXXAAGGGCACCACAAGAACGC-3′) | | |
|  | s15 (5'- XXXXXXXXCCTATCACATAATCATGAAAG-3') | | |
| Thermal cycling | 1 cycle | 94°C | 1 min |
|  | 60 cycles | 94°C | 30 sec |
|  |  | 52°C | 30 sec |
|  |  | 72°C | 30 sec |
|  | 1 cycle | 72°C | 2 min |

* Sequences of the primers used to amplify foraminifera-specific 37f hypervariable region of 18S rRNA gene ^4^

| Primer name | Primer sequence (5' - 3') | Primer name | Primer sequence (5' - 3') |
| --- | --- | --- | --- |
| s14F1-A | CACACACAAAGGGCACCACAAGAACGC | s15-A | AGACACACCCACCTATCACAYAATCATG |
| s14F1-B | CACATGATAAGGGCACCACAAGAACGC | s15-B | AGATGATGCCACCTATCACAYAATCATG |
| s14F1-C | CACTGATCAAGGGCACCACAAGAACGC | s15-C | AGTCGCGTCCACCTATCACAYAATCATG |
| s14F1-D | CAGCGTACAAGGGCACCACAAGAACGC | s15-D | AGTGCTGCCCACCTATCACAYAATCATG |
| s14F1-E | CAGTCGTAAAGGGCACCACAAGAACGC | s15-E | ATAGACTGCCACCTATCACAYAATCATG |
| s14F1-F | CGATCTACAAGGGCACCACAAGAACGC | s15-F | ATATCTACCCACCTATCACAYAATCATG |
| s14F1-G | CGATGACAAAGGGCACCACAAGAACGC | s15-G | ATGCGTAGCCACCTATCACAYAATCATG |
| s14F1-H | CGCACGTCAAGGGCACCACAAGAACGC | s15-H | ATGTCAGTCCACCTATCACAYAATCATG |
| s14F1-I | GACTGTATAAGGGCACCACAAGAACGC | s15-I | ATGTGCTCCCACCTATCACAYAATCATG |
| s14F1-J | GAGACTCTAAGGGCACCACAAGAACGC | s15-J | CAGTGCAGCCACCTATCACAYAATCATG |
| s14F1-K | GAGAGATAAAGGGCACCACAAGAACGC | s15-K | CATCGTACCCACCTATCACAYAATCATG |
| s14F1-L | GCACTGCAAAGGGCACCACAAGAACGC | s15-L | CATGCAGTCCACCTATCACAYAATCATG |
| s14F1-M | GCATCATCAAGGGCACCACAAGAACGC | s15-M | CGACGAGCCCACCTATCACAYAATCATG |
| s14F1-N | GCGATGTCAAGGGCACCACAAGAACGC | s15-N | CGAGACGTCCACCTATCACAYAATCATG |
| s14F1-O | GCGTGTCAAAGGGCACCACAAGAACGC | s15-O | CGAGCTAGCCACCTATCACAYAATCATG |
| s14F1-P | TACTCGACAAGGGCACCACAAGAACGC | s15-P | CTAGCATCCCACCTATCACAYAATCATG |
| s14F1-Q | TAGATGCAAAGGGCACCACAAGAACGC | s15-Q | CTGCACGCCCACCTATCACAYAATCATG |
| s14F1-R | TAGCTATCAAGGGCACCACAAGAACGC | s15-R | CTGTATATCCACCTATCACAYAATCATG |
| s14F1-S | TAGTGACTAAGGGCACCACAAGAACGC | s15-S | TAGCATGTCCACCTATCACAYAATCATG |
| s14F1-T | TCACGATAAAGGGCACCACAAGAACGC | s15-T | TAGTGAGCCCACCTATCACAYAATCATG |
| s14F1-U | TCGTCTATAAGGGCACCACAAGAACGC | s15-U | TATCGCTGCCACCTATCACAYAATCATG |
| s14F1-V | TGACGTCTAAGGGCACCACAAGAACGC | s15-V | TATGACATCCACCTATCACAYAATCATG |
| s14F1-W | TGACTGACAAGGGCACCACAAGAACGC | s15-W | TGATATGCCCACCTATCACAYAATCATG |
| s14F1-X | TGCAGTACAAGGGCACCACAAGAACGC | s15-X | TGATGCATCCACCTATCACAYAATCATG |
| s14F1-Y | TGCATATAAAGGGCACCACAAGAACGC | s15-Y | TGTCATAGCCACCTATCACAYAATCATG |
| s14F1-Z | TGCTCTCAAAGGGCACCACAAGAACGC | s15-Z | TGTGCATGCCACCTATCACAYAATCATG |

**Table S4.** Sequencing data statistics and quality control results

| Name | Method | Raw paired-end reads | Raw reads | ASVs | Retained reads | Retained ASVs |
| --- | --- | --- | --- | --- | --- | --- |
| EDG1 | Sieved | 199,663 | 195,196 | 192 | 185,515 | 141 |
| EDG2 |  | 377,126 | 358,537 | 365 | 358,023 | 330 |
| EDG3 |  | 403,247 | 385,467 | 538 | 384,588 | 497 |
| IS1 |  | 358,591 | 340,383 | 460 | 331,059 | 422 |
| IS2 |  | 403,475 | 381,449 | 586 | 375,557 | 483 |
| NAL4 |  | 266,984 | 259,027 | 225 | 256,796 | 189 |
| NAL5 |  | 334,649 | 319,765 | 460 | 314,457 | 390 |
| NAL6 |  | 273,959 | 256,110 | 573 | 249,021 | 490 |
| NAL7 |  | 411,847 | 353,112 | 644 | 338,512 | 493 |
| RIJ1 |  | 404,865 | 372,858 | 510 | 371,198 | 475 |
| RIJ2 |  | 364,483 | 346,437 | 529 | 343,541 | 477 |
| RIJ3 |  | 381,675 | 362,406 | 519 | 359,640 | 493 |
| RIJ4 |  | 424,620 | 383,727 | 669 | 379,638 | 579 |
| WIJ1 |  | 209,741 | 192,331 | 329 | 190,445 | 267 |
| WIJ3 |  | 342,889 | 329,614 | 461 | 323,280 | 381 |
| Mean of Sieved | | **343,854** | **322,427** | **471** | **317,418** | **407** |
| EDG1 | Unsieved | 56,411 | 54,912 | 108 | 50,894 | 84 |
| EDG2 |  | 44,506 | 41,057 | 188 | 41,038 | 186 |
| EDG3 |  | 93,269 | 86,133 | 395 | 85,632 | 382 |
| IS1 |  | 44,606 | 42,066 | 332 | 41,788 | 322 |
| IS2 |  | 101 | 53 | 20 | 53 | 20 |
| NAL4 |  | 42,053 | 37,505 | 227 | 36,735 | 215 |
| NAL5 |  | 63,713 | 54,901 | 333 | 54,172 | 311 |
| NAL6 |  | 49,556 | 46,896 | 441 | 45,918 | 424 |
| NAL7 |  | 58,636 | 52,677 | 428 | 41,462 | 388 |
| RIJ1 |  | 50,276 | 45,392 | 414 | 45,071 | 405 |
| RIJ2 |  | 66,043 | 61,872 | 304 | 61,778 | 300 |
| RIJ3 |  | 69,189 | 64,669 | 412 | 64,050 | 403 |
| RIJ4 |  | 44,368 | 41,456 | 439 | 40,956 | 411 |
| WIJ1 |  | 67,597 | 56,299 | 228 | 56,241 | 219 |
| WIJ3 |  | 60,648 | 56,948 | 330 | 56,442 | 311 |
| Mean of Unsieved | | **57,919** | **53,056** | **327** | **51,584** | **312** |

**Table S5**: Total number of ASVs and sequence read abundance identified within the foraminiferal 37f dataset.

**Table S6.** Permutational multivariate analysis of variance (PERMANOVA) results based on Bray-Curtis dissimilarities using *adonis* procedure for showing the effects of method, site, depth and their interaction on the foraminiferal community structure.

| Source of variation | Df | Sum of Sqs | R2 | pseudo-F | Pr(>F) |
| --- | --- | --- | --- | --- | --- |
| Method | 1 | 0.216 | 0.03767 | 1.7011 | 0.061 |
| Site | 4 | 2.0593 | 0.35916 | 4.0546 | 0.001 (***) |
| Depth | 1 | 0.5242 | 0.09142 | 4.1283 | 0.001 (***) |
| Method × Site | 4 | 0.2825 | 0.04927 | 0.5562 | 0.999 |
| Method × Depth | 1 | 0.0623 | 0.01086 | 0.4903 | 0.977 |
| Site × Depth | 4 | 1.1464 | 0.19994 | 2.2571 | 0.002 (**) |
| Method × Site × Depth | 3 | 0.1734 | 0.03024 | 0.4551 | 1 |
| Residual | 10 | 1.2697 | 0.22145 |  |  |
| Total | 28 | 5.7336 | 1 |  |  |

Df: degrees of freedom; Sum of Sqs —sum of squares; R2: the effect size; pseudoF - F value by permutation, Pr(>F): level of significance based on 999 random permutations. Significance codes: 0 ‘***’, 0.001 ‘**’, 0.01 ‘*’.

**Table S7.** Correlation of environmental variables with foraminiferal community compositions following *envfit* procedure. Pr (>r) indicates the level of significance based on 999 random permutations. Significance codes: 0 ‘***’, 0.001 ‘**’, 0.01 ‘*’.

| Variables | r2 | Pr(>r) |
| --- | --- | --- |
| Vectors |  |  |
| Depth | 0.6248 | 0.001 (***) |
| Temperature surface | 0.2077 | 0.055 |
| Temperature bottom | 0.1634 | 0.102 |
| Salinity surface | 0.1309 | 0.205 |
| Salinity bottom | 0.8513 | 0.001 (***) |
| Turbidity surface | 0.6633 | 0.001 (***) |
| Turbidity bottom | 0.8308 | 0.001 (***) |
| Factors |  |  |
| Method | 0.0010 | 0.9580 |
| Site | 0.4551 | 0.002 (**) |

**Table S8.** Foraminiferal ASVs and their correlations as computed using sparse PLS regression (sPLS). Correlations between foraminiferal ASVs and environmental parameters are revealed using standard methods for regression-based modelling of high dimensional data. Environmental parameters correspond to Depth, Bottom Salinity, Surface Temperature, Surface Salinity, Bottom Temperature, Bottom Turbidity, and Surface Turbidity.

**Reference**

1 Cottier, F. *et al.* Water mass modification in an Arctic fjord through cross-shelf exchange: The seasonal hydrography of Kongsfjorden, Svalbard. *Journal of Geophysical Research: Oceans* **110**, doi:<https://doi.org/10.1029/2004JC002757> (2005).

2 Lejzerowicz, F., Esling, P. & Pawlowski, J. Patchiness of deep-sea benthic Foraminifera across the Southern Ocean: Insights from high-throughput DNA sequencing. *Deep Sea Research Part II: Topical Studies in Oceanography* **108**, 17-26, doi:<https://doi.org/10.1016/j.dsr2.2014.07.018> (2014).

3 Barrenechea Angeles, I. *et al.* Planktonic foraminifera eDNA signature deposited on the seafloor remains preserved after burial in marine sediments. *Sci Rep* **10**, 20351, doi:10.1038/s41598-020-77179-8 (2020).

4 Esling, P., Lejzerowicz, F. & Pawlowski, J. Accurate multiplexing and filtering for high-throughput amplicon-sequencing. *Nucleic Acids Research* **43**, 2513-2524, doi:10.1093/nar/gkv107 (2015).
